# Association of preconception mixtures of phenol and phthalate metabolites with birthweight among subfertile couples

**DOI:** 10.1097/EE9.0000000000000222

**Published:** 2022-08-31

**Authors:** Yu Zhang, Vicente Mustieles, Paige L. Williams, Irene Souter, Antonia M. Calafat, Melina Demokritou, Alexandria Lee, Stylianos Vagios, Russ Hauser, Carmen Messerlian

**Affiliations:** a Department of Environmental Health, Harvard T.H. Chan School of Public Health, Boston, Massachusetts; b University of Granada, Center for Biomedical Research (CIBM), Instituto de Investigación Biosanitaria Ibs GRANADA, Consortium for Biomedical Research in Epidemiology and Public Health (CIBERESP), Granada, Spain; c Department of Epidemiology, Harvard T.H. Chan School of Public Health, Boston, Massachusetts; d Department of Biostatistics, Harvard T.H. Chan School of Public Health, Boston, Massachusetts; e Massachusetts General Hospital Fertility Center, Boston, Massachusetts; f National Center for Environmental Health, Centers for Disease Control and Prevention, Atlanta, Georgia.

**Keywords:** phthalate, phenol, preconception, paternal, birthweight, singleton

## Abstract

Although parental preconception exposure to some phenols and phthalates have been associated with reduced birthweight, few studies have examined these chemicals as complex mixtures.

**Methods:**

We included 384 mothers and 211 fathers (203 couples) who gave birth to 384 singletons from a prospective cohort of couples seeking fertility evaluation. Urinary concentrations of bisphenol A (BPA), parabens, and 11 phthalate metabolites including those of di(2-ethylhexyl) phthalate (DEHP) were examined. Birthweight was abstracted from delivery records. We used principal component analysis and Bayesian Kernel Machine Regression (BKMR) to examine maternal and paternal preconception mixtures in relation to singleton birthweight. We also fit couple-based BKMR with hierarchical variable selection to assess couples’ joint mixtures in relation to birthweight.

**Results:**

PC scores of maternal and paternal preconception low molecular weight phthalates factor, and paternal preconception DEHP-BPA factor were associated with reduced birthweight. In BKMR models, we found that maternal preconception monoethyl phthalate and BPA concentrations, and paternal preconception mono-n-butyl phthalate concentrations were inversely associated with birthweight when the remaining mixture components were held at their median concentrations. In couple-based BKMR models, paternal preconception biomarkers contributed more to couples’ joint effect on birthweight compared with maternal preconception biomarkers. A decreasing trend of birthweight was observed across quantiles of maternal, paternal, and couples’ total preconception mixture concentrations, respectively.

**Conclusions:**

Results from this preconception cohort of subfertile couples suggest a complex interplay between paternal and maternal preconception exposure to mixtures of nonpersistent chemicals, with both parental windows of exposure jointly contributing to reduced birthweight.

**What this study adds**

Investigating mixtures of environmental chemicals is a crucial research area highlighted by the NIEHS as a strategic priority. Our mixtures analyses identified real-world exposure patterns of select phenols and phthalates, and jointly examined these mixture groups in both parents in relation to birth weight. Our results suggest that birthweight is influenced by both parents’ preconception exposure and paternal preconception exposure might play a more important role. Given the often-overlooked paternal preconception period, this study has important implications for couple-based reproductive health interventions and policies.

## Introduction

Birthweight is an important marker of the intrauterine environment^1,2^ and has long-term effects on adult health and disease.^3-8^ Low birthweight (<2500 g) is one of the strongest predictors of neonatal health and survival.^9^ In 2015, over 20 million (14.6%) babies globally were born low birthweight.^10^ Although many determinants of low birthweight have been recognized, including maternal nutritional status, tobacco use, prepregnancy weight, and gestational weight gain, others remain unknown.^11,12^ Increasing evidence highlights the potential role of environmental exposures on birthweight, including exposure to nonpersistent endocrine disrupting chemicals.^13-16^

Phthalates and phenols are endocrine disrupting chemicals widely used in cosmetics, food-packaging materials, plastics, and numerous other consumer products, resulting in ubiquitous human exposure.^17,18^ Several compounds from both chemical families have raised concerns over potential reproductive and developmental toxicities,^19-23^ including intrauterine growth restriction in mice.^24,25^ Several epidemiologic studies have reported inverse associations between prenatal phenol and phthalate metabolite concentrations and fetal growth and birthweight.^15,26-31^ Moreover, previous studies have identified the preconception period as a vulnerable but underexplored window of maternal and paternal exposure to phenol and phthalate biomarkers in relation to singleton birthweight.^32-35^ Epigenetic modifications in the gametes of both parents during the pre- or periconception period may explain these observed associations.^36-39^

Studying chemical mixtures in relation to health outcomes in human populations is one of the current challenges in environmental epidemiology.^40^ Humans are exposed to dozens of chemical families simultaneously, and environmental chemicals within such mixtures may interact with each other leading to additive, synergistic, or even antagonistic effects.^41-45^ Phenols and phthalates share common exposure sources, including diet (e.g., packaged food), cosmetics, and other personal care products, leading to potential coexposure.^46-48^ The possibility of such combined effects of phenol and phthalate mixtures in human populations is supported by existing toxicological data and warrants further confirmation in well-conducted epidemiologic studies.^49-51^

In previous work using the same cohort, we reported that maternal and paternal preconception urinary phenol and phthalate metabolite concentrations were associated with reduced birthweight.^32-34^ Previous evaluations also demonstrated associations of prenatal phthalate mixtures with reduced birthweight.^52^ Thus, given the high potential for coexposure to mixtures of phenols and phthalates and the possibility of converging effects, this study aimed to examine maternal and paternal preconception urinary concentrations of phenol and phthalate mixtures on singleton birthweight.

## Methods

### Study cohort

The Environment and Reproductive Health (EARTH) Study is a prospective preconception cohort of couples recruited from the Massachusetts General Hospital Fertility Center between 2005 and 2018. The study aimed to examine how environmental and nutritional factors from preconception throughout pregnancy in both males and females influence fertility, pregnancy, and perinatal outcomes. Details of the cohort can be found elsewhere.^53^ Women 18–46 and men 18–55 years of age were eligible to participate independently or as a couple. General and lifestyle questionnaires were completed at study enrollment. Participants underwent anthropometric measurements, and provided a spot urine and blood sample at baseline, and then subsequently during each fertility treatment cycle, as well as across pregnancy trimesters for those achieving conception.

The present study included 384 females and 211 males (203 couples) who gave birth to a singleton infant between 2005 and 2018 and for whom we had at least one urine sample collected before conception of the index birth quantified for phenol and phthalate biomarkers. Study details were explained to participants by trained study staff before obtaining signed informed consents. The study was approved by the Institutional Review Boards of Massachusetts General Hospital, Harvard T.H. Chan School of Public Health, and the Centers for Disease Control and Prevention (CDC).

### Exposure assessment

A spot urine sample was obtained at study entry for each participant. Females provided up to two additional spot urine samples per fertility treatment cycle with the first sample collected during the follicular phase of the cycle and the second obtained on the day of the fertility procedure [at time of oocyte retrieval, embryo transfer for fresh or frozen in vitro fertilization (IVF) treatment, or on the day of intrauterine insemination (IUI)]. In addition to the baseline samples obtained at study entry, males provided one additional spot urine sample per cycle on the day when their female partner underwent the fertility procedure.

Urine was collected in a polypropylene specimen cup and analyzed for specific gravity (SG) with a handheld refractometer (National Instrument Company, Inc., Baltimore, MD, USA). Urine samples were divided into aliquots, frozen for long-term storage at –80°C, and then shipped on dry ice overnight to the CDC (Atlanta, GA, USA) for quantification of urinary phenol and phthalate metabolite concentrations using solid phase extraction coupled with high-performance liquid chromatography-isotope dilution tandem mass spectrometry.^54^ The urinary concentrations of four phenols and eleven phthalates were quantified. Phenols included bisphenol A (BPA), methylparaben, propylparaben, and butylparaben. Phthalates included monoethyl phthalate (MEP); mono-n-butyl phthalate (MBP); mono-isobutyl phthalate (MiBP); monobenzyl phthalate (MBzP); mono(3-carboxypropyl) phthalate (MCPP); monocarboxyisooctyl phthalate (MCOP); monocarboxyisononyl phthalate (MCNP); and four di-(2-ethylhexyl) phthalate (DEHP) metabolites: mono(2-ethylhexyl) phthalate (MEHP), mono(2-ethyl-5-hydroxyhexyl) phthalate (MEHHP), mono(2-ethyl-5-oxohexyl) phthalate (MEOHP), and mono(2-ethyl-5-carboxypentyl) phthalate (MECPP). The limits of detection (LOD) for biomarker concentrations ranged from 0.1 to 1.2 ng/mL. Concentrations below the LOD were assigned the LOD divided by the square root of two.^55^

### Outcome assessment

Trained study staff abstracted birthweight (g) from delivery records. Implausible birthweight values were assessed by examining corresponding gestational age and then cross-validating with delivery records (gold standard). Gestational age was also abstracted from delivery records and validated using the American College of Obstetricians and Gynecologists (ACOG) guidelines for dating births following medically assisted reproduction.^56^

### Covariates

Paternal and maternal age, education, race, and smoking status were obtained by self-reported questionnaires. The height and weight of participants were measured by a study staff at study entry. Body mass index (BMI) was calculated as kg/m^2^. The underlying cause of infertility was diagnosed by the treating infertility physician using the Society for Assisted Reproductive Technology (ART) definitions.^57,58^ Type of medically assisted reproduction used in the cycle of conception was abstracted from electronic medical records by trained study staff. Mode of conception was dichotomized as: ART (all IVF protocols, including intracytoplasmic sperm injection) versus non-ART (all IUI or ovarian stimulation protocols and nonmedically assisted/naturally conceived) conceptions.

### Statistical analyses

In order to account for urinary dilution, each biomarker concentration was multiplied by [(SGp-1)/(SGi-1)], where SGi is the SG of the participant’s sample and SGp is the mean SG for all male (mean = 1.016) or all female (mean = 1.015) participants included in the study.^59^ SG-adjusted biomarker concentrations were then natural log-transformed to standardize the distribution and reduce the influence of extreme values. The mean preconception biomarker concentration was estimated by averaging the natural log-transformed concentrations of the multiple urine samples collected per participant, that is, those obtained from study entry up to and including the sample from the cycle of conception of the index pregnancy.

We calculated descriptive statistics for biomarker concentrations and examined the Spearman correlation coefficients between biomarker concentrations for maternal and paternal preconception windows respectively, as well as for biomarker concentrations within couples. The clinical and demographic characteristics of study participants and their singletons were summarized as mean (SD) or number (%).

We first used principal component analysis (PCA) with varimax rotation to group phenols and phthalates biomarkers into uncorrelated components based on their correlations. Factors with eigenvalues greater than one were identified as principal components (PCs).^60^ We then fitted multivariable linear regression models for the PCA-derived scores of PCs (entered simultaneously) and birthweight to calculate the adjusted difference (beta coefficient) and 95% confidence interval (95% CI) in birthweight (g) per unit increase in each PC score, controlling for the rest of PCs. To make the results of PCA regressions more intuitive and to assess non-linearity, we also fitted multivariable linear regression models across quartiles of PCA-derived PC scores for the most relevant findings. We conducted PCA and linear regressions separately for both maternal and paternal preconception windows.

Second, we employed Bayesian Kernel Machine Regression (BKMR) with a Gaussian kernel exposure-response function to examine the associations between individual phenol and phthalate biomarker concentrations and birthweight in the context of mixtures, as well as the effect of the total maternal and paternal mixture on birthweight. BKMR can flexibly model the mixtures’ joint effect accounting for the correlations and interactions among each mixture component.^61^ We utilized hierarchical variable selection within the BKMR models based on PCA results, by grouping biomarkers according to the components of PCA-derived factors.

We calculated the group posterior inclusion probability (groupPIP) and conditional posterior inclusion probability (condPIP) for BKMR models. GroupPIPs represent the probability of including a particular biomarker group in the model and condPIP represent the probability of including a particular biomarker within its biomarker group.

We present BKMR results as: (1) univariate exposure-response associations between individual natural log-transformed biomarker concentrations and birthweight when holding other biomarkers in the mixture at their median concentrations; (2) potential interactions between biomarkers by estimating the change in birthweight comparing each individual biomarker concentration at 25th to 75th percentiles, when setting the remaining biomarker concentrations at their 25th, 50th, or 75th percentiles, respectively; and (3) the joint effect of the total mixture on birthweight by examining the change in birthweight when comparing all biomarkers at their median concentrations (reference) to their concentrations ranging from the 25th to the 75th percentile.

Covariates were selected *a priori* as potential confounders based on current knowledge using a directed acyclic graph (DAG). Covariates for maternal preconception exposure included maternal age and BMI (continuous), maternal education (<college, college, graduate degree), smoking status (never smoked, ever smoked), race (Caucasian, Black/African American, Asian, Other), and mode of conception of index pregnancy (ART, non-ART). Covariates for paternal preconception exposure included maternal and paternal age and BMI (continuous), maternal and paternal smoking (never smoked, ever smoked), maternal races (Caucasian, Black/African American, Asian, Other), maternal education (<college, college, graduate degree), and mode of conception (ART, non-ART). Because gestational age may be a causal intermediate between exposure to chemical mixtures and birthweight, we only adjusted for gestational age in a sensitivity analysis. We interpreted our results considering patterns of associations and concordance with previous epidemiological findings rather than depending solely on *P* values and statistical significance.^62^ Statistical analyses were conducted with SAS (version 9.4; SAS Institute Inc., Cary, NC), and BKMR models were conducted using R package bkmr.^63^

### Sensitivity analyses

To examine couples’ joint exposure to phenol and phthalate mixtures in relation to birthweight, we restricted the BKMR model to the 203 couples. We included couples preconception mixture biomarkers in the same model, and separated them into maternal and paternal preconception groups in the hierarchical variable selection in order to compare the parent-of-origin contribution (reflected as group PIPs in the BKMR model) to the study outcome.^64^ We also restricted the maternal preconception analyses to the 203 women with a male partner in the study to obtain more comparable results across maternal and paternal models with the same sample size. We further adjusted for gestational age in all models as a means of comparing models with and without this potential causal intermediate.

## Results

### Study cohort

A total of 384 mothers and 211 fathers (203 couples) were included in the study with a mean age of 35 and 36 years and a mean BMI of 24 and 28 kg/m^2^, respectively (Table 1). For the 384 singleton infants, mean (SD) gestational age was 39 (1.7) weeks and 8% (n = 30) were born preterm (Table 2). Females who enrolled with their male partner (n = 203) were more likely to have a male factor infertility diagnosis at baseline (28% vs. 18%) and have slightly higher geometric mean BPA of sum of DEHP metabolites concentrations (41 ng/mL vs. 38 ng/mL) compared with females who enrolled without a partner (n = 181) (eTable 1; http://links.lww.com/EE/A196).

**Table 1.** Parental characteristics from 384 women and 211 men participating in the EARTH study, 2005–2018.

Parental characteristic	Maternal	Paternal
	N = 384	N = 211
Age (yrs)		
Mean (SD)	34.6 (4.0)	35.8 (4.6)
Age > 35, n (%)	157 (41)	114 (54)
Race, n (%)		
White	323 (84)	186 (88)
Black	10 (3)	4 (2)
Asian	35 (9)	14 (7)
Other	16 (4)	7 (3)
BMI (kg/m^2^)		
Mean (SD)	24 (4.1)	28 (6.3)
BMI >25, n (%)	120 (31)	144 (68)
Education, n (%)		
<College	50 (13)	71 (34)
College graduate	121 (32)	58 (27
Graduate degree	213 (55)	78 (37)
Missing	n/a	4 (2)
Smoking status, n (%)		
Never	289 (75)	145 (69)
Ever (former or current)	95 (25)	66 (31)
Infertility diagnosis, n (%)		
Male factor	90 (23)	65 (31)
Female factor	122 (32)	61 (29)
Unexplained	172 (45)	85 (40)
Primiparous, n (%)	320 (83)	n/a

**Table 2.** Birth characteristics of 384 singletons from the EARTH study, 2005–2018.

Child characteristics	Births	
	2005–2018	
	N = 384	N = 203^a^
Male, n (%)	195 (51)	100 (49)
Birthweight (g)		
Mean (SD)	3,357 (536)	3,353 (497)
Min-max	1,310–4,790	1,850–4,790
Low birthweight		
<2500 g, n (%)	18 (5)	6 (3)
Gestational age at birth		
Mean, wks (SD)	39 (1.7)	39 (1.5)
Min-max	29–42	33–42
Preterm birth		
<37 wks, n (%)	30 (8)	14 (7)
Mode of conception, n (%)		
ART^b^	223 (58)	126 (62)
Non-ART^c^	161 (42)	77 (38)

^a^Singletons from couples participating in the study.

^b^ART: fresh or frozen in vitro fertilization protocols, including intracytoplasmic sperm injection.

^c^ Non-ART: intrauterine insemination with or without ovulation induction/stimulation; ovulation induction/stimulation with timed intercourse, or nonmedically assisted/naturally conceived.

### Urinary biomarker concentrations

We included 1600 maternal preconception and 557 paternal preconception urine samples in the present study. On average, women provided 4.2 (median: 3; IQR: 2–5) and men 2.6 (median: 2; IQR: 2–3) urine samples. Biomarker distributions and detection frequencies are presented in eTable 2; http://links.lww.com/EE/A196. Among the biomarkers examined, butylparaben had the lowest urinary detection frequency (63% for maternal preconception and 32% for paternal preconception). The remaining biomarkers had detection frequencies greater than or equal to 70%. eFigures 1–3; (http://links.lww.com/EE/A196) present Spearman correlation coefficient matrixes for maternal, paternal, and couples’ biomarker concentrations, respectively. Spearman correlation coefficients ranged from –0.15 (MEHP and MCOP) to 0.98 (MEOHP and MEHHP) for maternal preconception biomarkers (eFigure 1; http://links.lww.com/EE/A196), –0.06 (MEHP and MCOP) to 0.98 (MEOHP and MEHHP) for paternal preconception biomarkers (eFigure 2; http://links.lww.com/EE/A196), and –0.32 (maternal MEHHP and paternal MCOP) to 0.60 (maternal and paternal MECPP) for couples’ biomarker concentrations (eFigure 3; http://links.lww.com/EE/A196).

### Maternal preconception window

The PCA for the maternal preconception window identified four PCs named as DEHP-BPA factor, paraben factor, high molecular weight phthalate (HMWP) factor, and low molecular weight phthalate (LMWP) factor (eTable 3; http://links.lww.com/EE/A196). The DEHP-BPA factor accounted for 33% of the total mixture variance and showed high loading scores for MEOHP, MEHHP, MECPP, MEHP (the four DEHP metabolites measured) and BPA. The paraben factor accounted for 16% of the total variance and showed high loading scores for methyl-, propyl-, and butylparaben, and MEP. The HMWP factor accounted for 13% of the total variance and showed high loading scores for MCOP, MCPP, and MCNP. The LMWP factor accounted for 10% of the total variance presenting high loading scores for MiBP, MBP, and MBzP. This factor was termed LMWP factor for simplicity although MBzP is a HMWP.

Multivariable linear regression showed that a 1-unit-increase in maternal preconception LMWP factor score was associated with a 51 g decrease in birthweight (95% CI = –105, 2), while no association was observed for the other three maternal preconception PCA-derived factors (Table 3). Quartile analyses showed that the second (beta = –184; 95% CI = –336, –33) and fourth (beta = –155; 95% CI = –308, –2) quartiles of maternal preconception LMWP factor score were associated with decreased birthweight (p-trend= 0.11) compared to the first quartile of LMWP factor score (Table 4).

**Table 3.** Adjusted difference (95% CI) in birthweight (g) by PCA-derived factors scores from 384 mothers and 211 fathers in the EARTH Study, 2005–2018.

PCA-derived factors	Maternal		Paternal	
	Beta (95% CI) ^a^	*P*	Beta (95% CI) ^b^	*P*
DEHP-BPA factor	–2.0 (–55, 52)	0.94	–63 (–134, 7)	0.08
Paraben factor	–18.6 (–73, 35)	0.50	16 (–52, 84)	0.64
High molecular weight phthalate factor	17 (–37, 71)	0.54	–49 (–116, 19)	0.16
Low molecular weight phthalate factor	–51 (–105, 2)	0.06	–73 (–141, –5)	0.04

^a^Adjusted for maternal age (continuous), BMI (continuous), ART (yes/no), smoking (ever/never), education (categorical), races (categorical).

^b^Adjusted for maternal and paternal age (continuous), maternal and paternal BMI (continuous), maternal and paternal smoking (ever/never), maternal education (categorical), maternal races (categorical), ART (yes/no).

**Table 4.** Adjusted difference (95% CI) in birthweight (g) by quartiles of PCA-derived factors scores from 384 mothers and 211 fathers in the EARTH Study, 2005–2018.

PCA-derived factors	Beta (95% CI)
Maternal LMWP factor score	
Q1	ref
Q2	–184 (–336, –33)
Q3	–109 (–260, 41)
Q4	–154 (–307, –2)
p for trend	0.11
Paternal DEHP-BPA factor score	
Q1	ref
Q2	–54 (–262, 155)
Q3	–222 (–431, 13)
Q4	–112 (–321, 98)
p for trend	0.16
Paternal HMWP factor score—+	
Q1	ref
Q2	50 (–147, 247)
Q3	12 (–187, 210)
Q4	–88 (–286, 111)
p for trend	0.21
Paternal LMWP factor score	
Q1	ref
Q2	–97 (–294, 99)
Q3	–86 (–283, 110)
Q4	–183 (–381, 16)
p for trend	0.13

^a^Adjusted for maternal age (continuous), BMI (continuous), ART (yes/no), smoking (ever/never), education (categorical), races (categorical).

^b^Adjusted for maternal and paternal age (continuous), maternal and paternal BMI (continuous), maternal and paternal smoking (ever/never), maternal education (categorical), maternal races (categorical), ART (yes/no).

**Figure 1.**
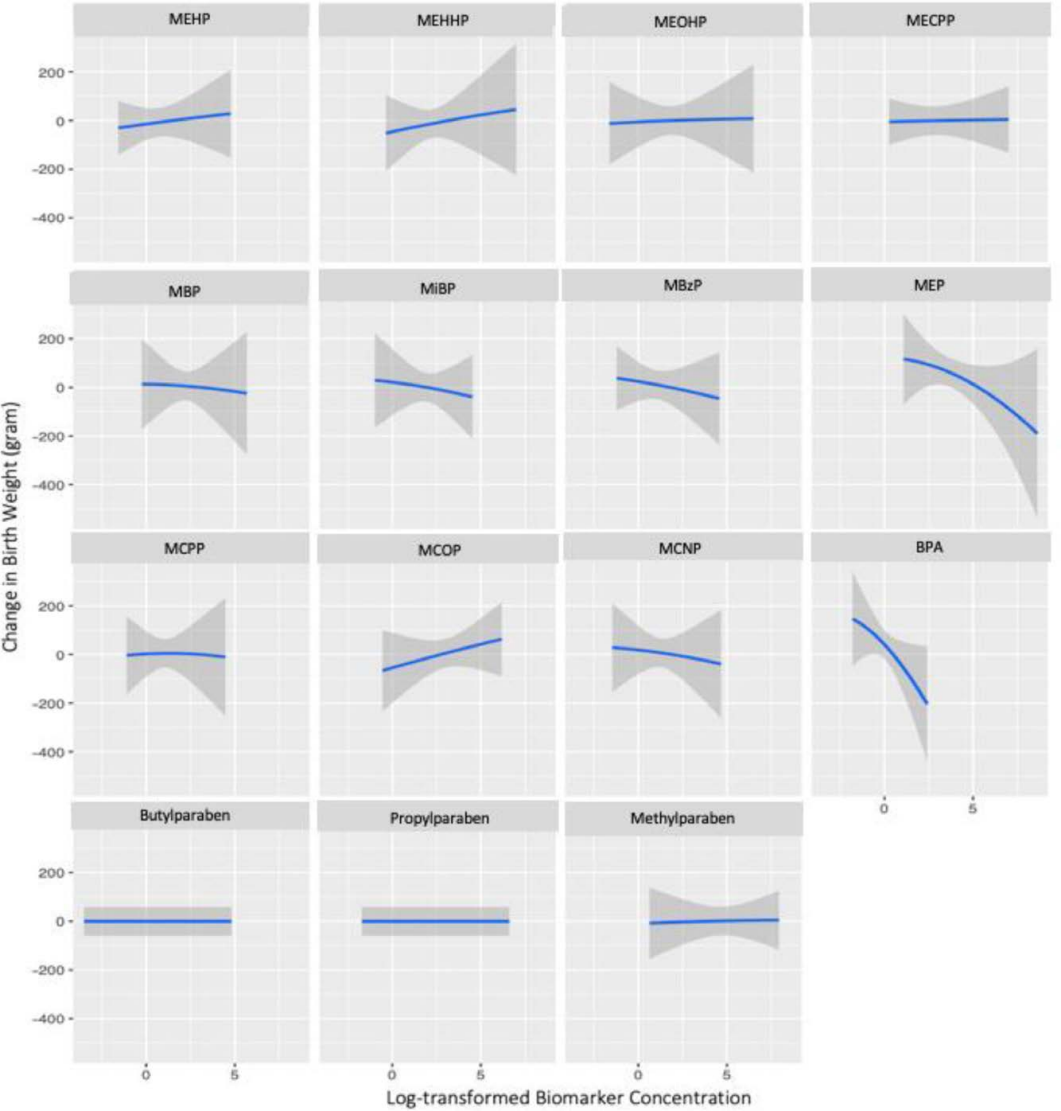
Dose-response associations of individual *maternal preconception* phenol and phthalate metabolite concentrations on birthweight, holding all other biomarkers at their median concentrations among 384 mothers in the Environment and Reproductive Health (EARTH) Study, 2005–2018. bisphenol A (BPA); methylparaben (MPB); propylparaben (PPB); butylparaben (BPB); monoethyl phthalate (MEP); mono-n-butyl phthalate (MBP); mono-isobutyl phthalate (MiBP); monobenzyl phthalate (MBzP); mono(2-ethylhexyl) phthalate (MEHP); mono(2-ethyl-5-hydroxyhexyl) phthalate (MEHHP); mono(2-ethyl-5-oxohexyl) phthalate (MEOHP); mono(2-ethyl-5-carboxypentyl) phthalate (MECPP); mono(3-carboxypropyl) phthalate (MCPP); monocarboxyisooctyl phthalate (MCOP); monocarboxyisononyl phthalate (MCNP); models were adjusted for maternal age (continuous), BMI (continuous), ART (yes/no), smoking (ever/never), education (categorical), races (categorical).

The maternal preconception BKMR model showed that the DEHP-BPA factor had the highest groupPIP (*P* = 0.25) and the condPIP of BPA was the highest (*P* = 0.90) within this group (eTable 4; http://links.lww.com/EE/A196). Univariate exposure-response analysis showed that maternal preconception urinary BPA and MEP concentrations were negatively associated with birthweight, respectively, when holding all other biomarkers at their median concentrations (Figure 1). A suggestive positive trend was found for maternal preconception MCOP concentration and birthweight (Figure 1). No obvious patterns were found for other biomarkers examined (Figure 1). In examining the interaction within maternal preconception biomarker mixture, we found no apparent differences in the associations between individual biomarkers at the 25th versus 75th percentiles and birthweight, comparing the results when concentrations of the remaining maternal biomarkers were set at their 25th, 50th, and 75th percentiles (eFigure 4; http://links.lww.com/EE/A196). A clear decreasing trend of birthweight across quantiles of the total maternal mixture was observed (eFigure 5; http://links.lww.com/EE/A196). Compared to the median concentration of the total maternal preconception mixture, the concentration at the 75th percentile was associated with a 50 g decrease (95% CI = –116, 17) in birthweight (eFigure 5; http://links.lww.com/EE/A196).

### Paternal preconception window

The PCA for paternal preconception window identified the same four PCs as the maternal preconception analysis, but showed differences in the percent of the total variance explained by each factor (eTable 5; http://links.lww.com/EE/A196). In the paternal preconception model, the DEHP-BPA factor still accounted for the highest amount of total variance (34%), while the HMWP, LMWP, and paraben factors accounted for 15%, 14% and 10% of the variance, respectively.

In multivariable linear models, a 1-unit increase in paternal DEHP-BPA factor and LMWP factor scores were associated with a 63 g (95% CI = –134, 7) and a 73 g (95% CI = –141, –5) decrease in birthweight, respectively (Table 3). The third quartile of paternal preconception DEHP-BPA factor score was associated with a 222 g decrease (95% CI = –431, 13) in birthweight compared to the first quartile (Table 4). A 183 g decrease (95% CI = –381, 15) in birthweight was found in the fourth quartile of the LMWP factor score compared to the first quartile (Table 4). A suggestive negative association between the paternal HMWP factor score and birthweight was observed (beta = –49, 95% CI = –116, 19) (Table 3) but no obvious trend was observed across quartiles (Table 4). No association was found for the paternal preconception paraben factor score in relation to birthweight (Table 3).

**Figure 2.**
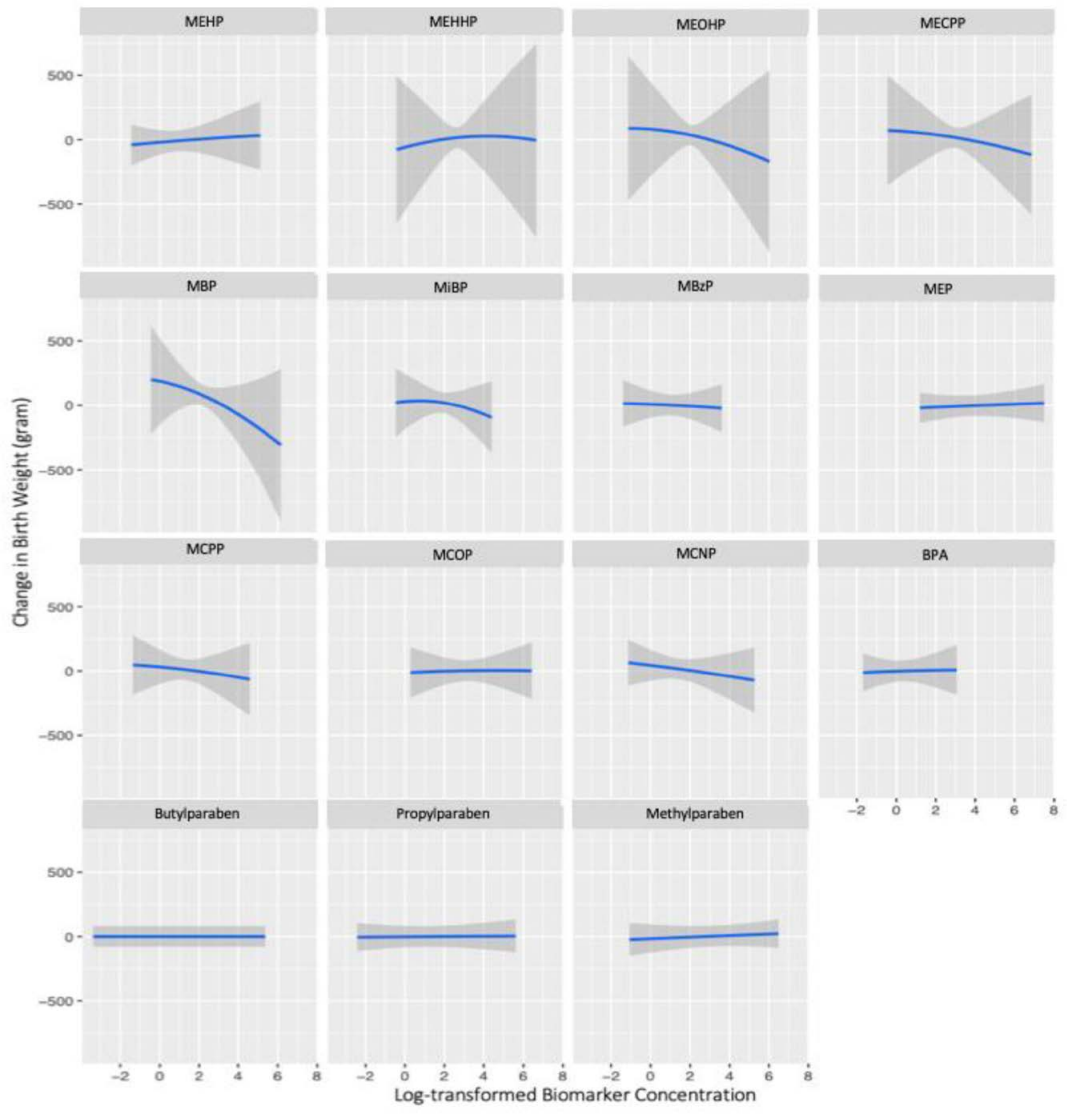
Dose-response associations of individual *paternal preconception* phenol and phthalate metabolite concentrations on birthweight, holding all other biomarkers at their median concentrations among 211 fathers in the Environment and Reproductive Health (EARTH) Study, 2005–2018. bisphenol A (BPA); methylparaben (MPB); propylparaben (PPB); butylparaben (BPB); monoethyl phthalate (MEP); mono-n-butyl phthalate (MBP); mono-isobutyl phthalate (MiBP); monobenzyl phthalate (MBzP); mono(2-ethylhexyl) phthalate (MEHP); mono(2-ethyl-5-hydroxyhexyl) phthalate (MEHHP); mono(2-ethyl-5-oxohexyl) phthalate (MEOHP); mono(2-ethyl-5-carboxypentyl) phthalate (MECPP); mono(3-carboxypropyl) phthalate (MCPP); monocarboxyisooctyl phthalate (MCOP); monocarboxyisononyl phthalate (MCNP); models were adjusted for maternal and paternal age (continuous), maternal and paternal BMI (continuous), maternal and paternal smoking (ever/never), maternal education (categorical), maternal races (categorical), ART (yes/no).

In the BKMR model for the paternal preconception window, LMWP factor had the highest groupPIP (*P* = 0.56) and MBP had the highest condPIP within this group (*P* = 0.66) (eTable 6; http://links.lww.com/EE/A196). In the univariate exposure-response analysis, we observed a negative association between MBP and birthweight when holding all other paternal biomarkers at their median concentrations (Figure 2). No apparent interactions were found for any paternal biomarker concentrations on birthweight (eFigure 6; http://links.lww.com/EE/A196). A significant decreasing trend was observed for birthweight across increasing quantiles of the total paternal preconception mixture (Figure 4). The paternal preconception mixture concentration at the 75th percentile was associated with 89 g decrease (95% CI = –175, –3) in birthweight compared to the median concentration (Figure 4). When setting the 25th percentile of the total paternal mixture concentration as the reference group, paternal preconception mixture concentrations at the 75th percentile were associated with a 169 g decrease (95% CI = –331, –7) compared to its 25th percentile (figure not shown).

**Figure 3.**
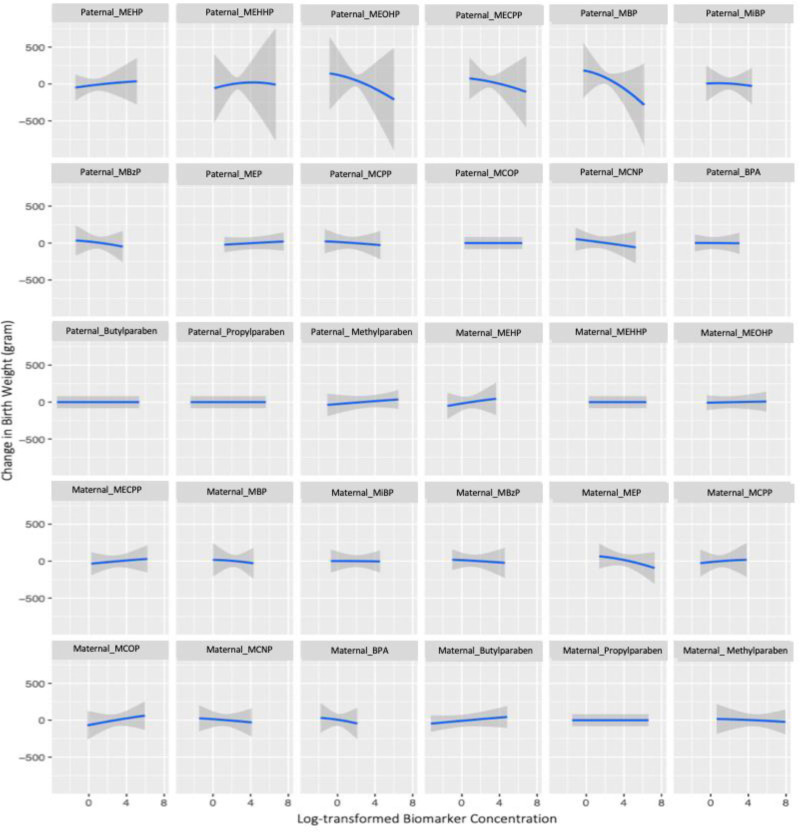
Dose-response associations of individual *couples’ preconception* phenol and phthalate metabolite concentrations on birthweight, holding all other biomarkers at their median concentrations among 203 couples in the Environment and Reproductive Health (EARTH) Study, 2005–2018. bisphenol A (BPA); methylparaben (MPB); propylparaben (PPB); butylparaben (BPB); monoethyl phthalate (MEP); mono-n-butyl phthalate (MBP); mono-isobutyl phthalate (MiBP); monobenzyl phthalate (MBzP); mono(2-ethylhexyl) phthalate (MEHP); mono(2-ethyl-5-hydroxyhexyl) phthalate (MEHHP); mono(2-ethyl-5-oxohexyl) phthalate (MEOHP); mono(2-ethyl-5-carboxypentyl) phthalate (MECPP); mono(3-carboxypropyl) phthalate (MCPP); monocarboxyisooctyl phthalate (MCOP); monocarboxyisononyl phthalate (MCNP); models were adjusted for maternal and paternal age (continuous), maternal and paternal BMI (continuous), maternal and paternal smoking (ever/never), maternal education (categorical), maternal races (categorical), ART (yes/no).

**Figure 4.**
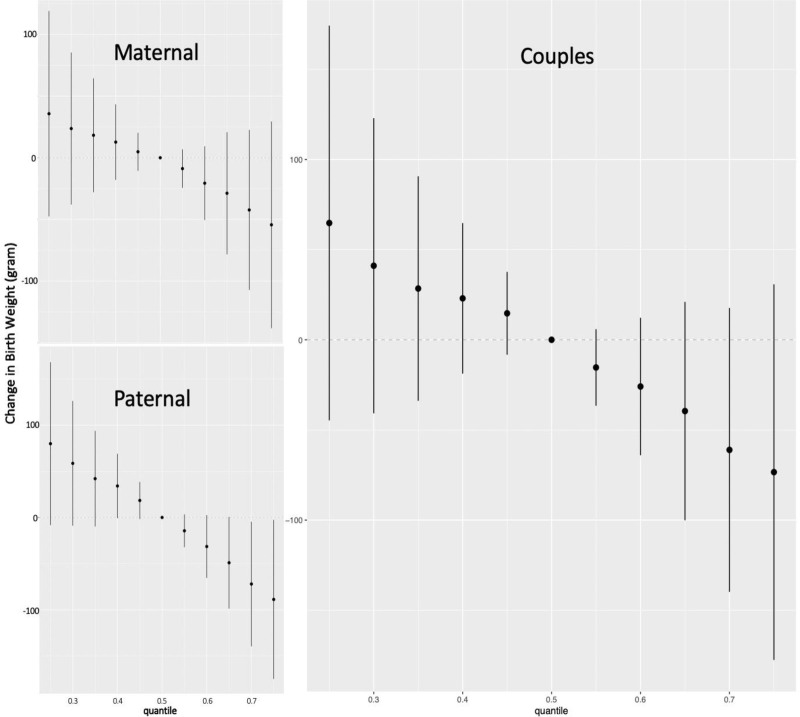
Joint effect (estimates and credible intervals) on birthweight comparing each 5th quantile change in total maternal, paternal, and couple’s preconception mixture concentrations from 25th to 75th quantile to the median concentration in the Environment and Reproductive Health (EARTH) Study, 2005–2018. Maternal model was restricted to women who participated as couples (n = 203), paternal model included all fathers (n = 211, i.e., 203 who participated as couples and 8 who participated independently), couples model included the 203 couples; Maternal model was adjusted for maternal age (continuous), BMI (continuous), ART (yes/no), smoking (ever/never), education (categorical), races (categorical); Paternal and couple’s model were adjusted for maternal and paternal age (continuous), maternal and paternal BMI (continuous), maternal and paternal smoking (ever/never), maternal education (categorical), maternal races (categorical), ART (yes/no).

### Sensitivity analyses

Couples’ BKMR model showed a much higher groupPIP of paternal preconception mixture (*P* = 0.72) compared to the maternal preconception mixture (*P* = 0.18) (eTable 7; http://links.lww.com/EE/A196). MBP had the highest condPIP within both the paternal mixture *P* = 0.41) and maternal mixture (*P* = 0.20) (eTable 7; http://links.lww.com/EE/A196). Paternal preconception MBP remained inversely associated with birthweight when the remaining maternal and paternal biomarker concentrations were held at their median concentrations (Figure 3). No obvious univariate dose-response relationships for individual maternal preconception biomarker and birthweight were observed when holding the rest of maternal and paternal biomarker concentrations at their medians (Figure 3). No interactions were discernible between couples’ biomarker concentrations and birthweight (eFigure 7; http://links.lww.com/EE/A196). We found a decreasing trend of birthweight across increasing quantiles of couples’ total mixture concentrations (Figure 4). A 73 g decrease (95% CI = –178, 31) in birthweight was found comparing couples’ total mixture concentration at the 75^th^ percentile to the median concentration (Figure 4). We further found a 138 g decrease (95% CI = –337, 60) in birthweight comparing the 75th percentile to the 25th percentile of couples’ total mixture concentration, when setting the 25th percentile as the reference group (figure not shown).

After further adjusting for gestational age in multivariable linear regressions, associations between maternal LMWP factor (β = –42; 95% CI = –84, 1), paternal DEHP-BPA factor (β = –49; 95% CI = –112, 13) and birthweight were attenuated slightly, while associations for paternal HMWP factor (β = –53; 95% CI = –112, 7) and LMWP factor (β = –82; 95% CI = –143, –22) were strengthened (eTable 8; http://links.lww.com/EE/A196). After adjusting for gestational age, the BKMR results, including univariates exposure-outcome association and the joint effect of the total mixture, did not meaningfully change for the maternal preconception BKMR models (eFigures 8 and 9; http://links.lww.com/EE/A196), attenuated for paternal preconception BKMR models (eFigures 10 and 11; http://links.lww.com/EE/A196), and strengthened for couples’ BKMR models (eFigures 12 and 13; http://links.lww.com/EE/A196). A significant decreasing trend was observed for birthweight across increasing quantiles of couples’ total mixture (eFigure 13; http://links.lww.com/EE/A196) comparable to the decrease reported in the primary analysis (Figure 4).

In models restricted to the 203 females who enrolled as couples, we found that maternal LMWP factor was associated with a 52 g decrease (95% CI = –122, 17) in birthweight after adjusting for covariates (eTable 9; http://links.lww.com/EE/A196). In BKMR models, LMWP factor - but not DEHP-BPA factor – had the highest though small groupPIP (*P* = 0.21), and MBP accounted for the highest condPIP within this group (*P* = 0.78) (eTable 10; http://links.lww.com/EE/A196). Apart from the associations of maternal BPA, MEP, and MCOP concentrations found in the overall cohort (Figure 1), a steep negative association was found for maternal MBP and MCNP and birthweight when holding the remaining maternal preconception biomarker concentrations at their medians (eFigure 14; http://links.lww.com/EE/A196). There was a consistent negative association between increasing quantiles of the total maternal preconception mixture, concentrations, and decreased birthweight (Figure 4).

## Discussion

In this preconception cohort of subfertile couples, we found that chemical mixtures containing LMWPs as the main component were associated with decreased birthweight in both maternal and paternal preconception windows. BKMR further identified that paternal MBP was negatively associated with birthweight when holding the remaining paternal biomarker concentrations at their median concentrations. Maternal preconception concentrations of BPA, MEP, and MBP were also negatively associated with birthweight, when the remaining maternal biomarker concentrations were held at their median concentrations. However, these univariate-response associations for maternal biomarkers were attenuated when considering both maternal and paternal (i.e., couples) biomarkers in the BKMR model. No interactions within mixtures in either maternal or paternal preconception models were observed. We found a clear negative joint effect of the total paternal preconception mixture on birthweight, while a suggestive negative trend for birthweight across quantiles of maternal preconception mixture. When considering the joint effect of couples’ mixtures in BKMR models, hierarchical variable selection showed a higher contribution of the paternal window on birthweight compared to maternal exposure. A clear negative joint association was found for couples’ total preconception exposure to phenol and phthalate mixtures in relation to birthweight.

Our mixture findings are consistent with previous studies using single-chemical analyses in the same cohort.^32-34^ Paternal preconception urinary concentrations of the four DEHP metabolites, MBP, MBzP, and MiBP were associated with decreases in birthweight among IVF-conceived singletons,^32^ while maternal preconception urinary BPA and MEP concentrations were associated with decreased birthweight among all singletons from the EARTH study.^32,33^ Only one additional cohort, the LIFE study, examined maternal and paternal preconception urinary concentrations of BPA and phthalate biomarkers in relation to birth outcomes.^35^ This study used single-chemical analyses and biomarker concentration was estimated using a single spot urine sample collected at study entry.^35^ No association was reported between maternal BPA and birthweight, but a decreasing trend for birthweight was found in the second compared to the first quartile of maternal MEP concentration after adjusting for paternal coexposure. The second quartile of paternal preconception MEHP concentration was also associated with decreased birthweight compared to the first quartile.^35^

The present study confirms previous results observed in single-chemical analyses, and additionally shows that (1) the total paternal preconception phenol and phthalate mixture was jointly associated with decreased birthweight; (2) couple’s total exposure to phenol and phthalate mixtures in the preconception period was also jointly associated with decreased birthweight; (3) the paternal window of exposure appeared to contribute more to the birthweight outcome than maternal preconception exposure to mixtures.

Although toxicological data are still scarce, preconception exposure to phenols and phthalates may influence male and female germlines through epigenetic modifications, which may persist in the embryo and contribute to adverse pregnancy outcomes.^36-39^ Our results suggest a higher contribution of paternal compared with maternal chemical mixtures on birthweight. Genomic imprinting is an epigenetic process that silences one parental allele, maternal or paternal, resulting in monoallelic expression.^65,66^ While paternally expressed genes tend to promote fetal growth, maternally expressed genes tend to suppress growth with the placenta playing a crucial role in these parental influences.^67^ Interestingly, paternally expressed genes seem to predominate in the placenta,^68^ and we previously observed that paternal preconception urinary concentrations of DEHP metabolites and MBP tended to be associated with lower placental weight in a sub-cohort analysis of the EARTH Study.^69^ Overall, we hypothesize that paternal chemical exposures before conception may interfere with the epigenetic programming of sperm, particularly imprinted genes, that may especially influence placental function predisposing to fetal growth restriction among other adverse pregnancy complications.^69-72^ Further mechanistic studies are needed to elucidate the contribution of maternal and paternal preconception environmental exposures to birth outcomes.

The joint effect of mixtures observed in our study is consistent with the so-called “cocktail effect”, a phenomenon by which mixtures of chemicals often show a heightened toxicity, even when each individual chemical is present at doses that do not produce any observable effect in isolation.^40^ Thus, it has been shown that concurrent administration of DEHP reduces the threshold at which BPA impairs blastocyst implantation in mice,^51^ and that a mixture of five chemicals including propylparaben, butylparaben, and DEHP, modulated the bioavailability and tissue distribution of BPA concentrations.^73^ Our data support low-dose mixture effects in human populations, which could be driven as a result of converging effects, and/or toxicokinetic modifications, because many phenols and phthalates are metabolized through common mechanisms.^74,75^

The current study has implemented two complementary statistical methods providing novel insights about the relationships between parental preconception exposure to phenol and phthalate mixtures and birthweight. We grouped mixtures using PCA ^60^ and examined which biomarker group derived from PCA was most relevant to the outcome. BKMR with hierarchical variable selection was able to identify, which biomarker group contributed more to the outcome and the most relevant specific individual component or metabolites inside each biomarker group, and it also examined shapes of the dose-response relationships for both individual biomarkers and the total mixtures on birthweight. A previous study of the same cohort utilized PCA and BKMR to examine prenatal exposure to phthalate mixture in relation to birthweight.^52^ Our study included a wider scope of environmental chemicals based on their common sources and examined both maternal and paternal exposures before conception. The use of hierarchical variable selection in the couples’ BKMR model allowed us to compare the relative contribution of maternal and paternal mixtures on birthweight. Another strength of this study was the opportunity to assess exposure prior to conception in both parents. Nevertheless, the study also has several limitations. First, our findings may not be directly generalizable to fertile couples because subfertile mothers and fathers could be more susceptible to environmental disruptions. Notwithstanding, our results are comparable to one preconception study in a non-subfertile population ^35^ and previous analyses on parental preconception exposure and preterm birth using the EARTH study were also consistent with findings from this non-subfertile cohort.^76,77^ Additionally, our results are compatible with several other prenatal studies investigating birth outcomes in fertile populations.^19,78^ Second, the short biological half-lives and episodic exposure patterns of these nonpersistent chemicals increase the likelihood of exposure misclassification. However, multiple repeated urine samples were collected thereby reducing exposure misclassification and its expected attenuation bias.^79^ Third, our mixtures analyses focused on nonpersistent chemicals used in the manufacture of plastics and in personal care products while human populations are normally exposed to multiple chemical families. Because of the inherent complexity of the investigated models, we did not consider potential exposure confounding during the prenatal window. Nevertheless, our previous single-chemical analyses in the same cohort did not show substantial prenatal exposure confounding.^32-34^

## Conclusion

Our results showed that maternal and paternal phenol and phthalate metabolite mixtures and couples’ exposure to total mixtures in the preconception period were associated with decreased birthweight. Among the complex patterns identified, paternal preconception MBP concentrations appeared to have the greatest negative influence on birthweight within the mixture. When maternal components within a mixture were considered, MEP and BPA appeared as the strongest contributors to reduced birthweight. Although additional preconception studies should confirm our findings, we found evidence that mixtures of nonpersistent chemicals can jointly influence birthweight, with different parent-of-origin contributions.

## Acknowledgments

We acknowledge all members of the EARTH Study team, including research staff Ramace Dadd and Myra Keller, and physicians and staff at Massachusetts General Hospital Fertility Center. We are grateful to all study participants and to Xiaoliu Zhou, Tao Jia, and Xiaoyun Ye (deceased) (CDC, Atlanta, GA) for measuring the urinary concentrations of phthalate and phenol biomarkers.

## Supplementary Material


