# Supplementary material for: Association of preconception mixtures of phenol and phthalate metabolites with birthweight among subfertile couples
Source: Environ Epidemiol. 2022 Aug 31;6(5):e222. doi: 10.1097/EE9.0000000000000222 (PMC9555928; doi:10.1097/EE9.0000000000000222)
Supplement: Supplementary file 1 [file ee9-6-e222-s001.docx]

**Association of Preconception Mixtures of Phenol and Phthalate Metabolites with Birthweight among Subfertle Couples**

**eTable 1.** Maternal characteristics of the 384 mothers and the 203 mothers who enrolled with male partner.

| Maternal Characteristic | All mothers | Mothers who enrolled with male partner |
| --- | --- | --- |
|  | N = 384 | N = 203 |
| Age (years) |  |  |
| Mean (SD) | 34.6 (4.0) | 34.3 (3.9) |
| Age>35, n (%) | 157 (41) | 79 (39) |
| Race, n (%) |  |  |
| White | 323 (84) | 167 (82) |
| Black | 10 (3) | 6 (3) |
| Asian | 35 (9) | 21 (10) |
| Other | 16 (4) | 9 (5) |
| Body Mass Index (BMI, Kg/m2) | | |
| Mean (SD) | 24.0 (4.1) | 24.0 (4.2) |
| BMI >25, n (%) | 120 (31) | 62 (31) |
| Education, n (%) | |  |
| < College | 50 (13) | 25 (12) |
| College Graduate | 121 (32) | 61 (30) |
| Graduate Degree | 213 (55) | 117 (58) |
| Smoking Status, n (%) | |  |
| Never | 289 (75) | 153 (75) |
| Ever (former or current) | 95 (25) | 50 (25) |
| Infertility Diagnosis, n (%) | | |
| Male Factor | 90 (23) | 64 (30) |
| Female Factor | 122 (32) | 61 (29) |
| Unexplained | 172 (45) | 85 (41) |
| Primiparous, n (%) | 320 (83) | 131 (84) |
| Maternal BPA concentration (ng/ml) |  |  |
| Geometric Mean (GSD) | 1.09 (0.04) | 1.12 (0.05) |
| Median | 1.05 | 1.1 |
| IQR 25th - 75th | 0.72-1.59 | 0.75 - 1.64 |
| Maternal ∑DEHP ^a^ concentration (ng/ml) |  |  |
| Geometric Mean (GSD) | 39.36 (1.78) | 40.83 (2.48) |
| Median | 34.08 | 35.58 |
| IQR 25th - 75th | 21.59 - 69.64 | 22.24 - 75.40 |

Note: BPA, Bisphenol A; DEHP, di-(2-ethylhexyl) phthalate; GSD, geometric standard deviation.

^a^: ∑DEHP concentration was calculated as the weighted molar sum of MEHP (molecular weight=272), MEHHP (molecular weight=294), MEOHP (molecular weight=292) and MECPP (molecular weight=308) concentrations expressed in µmol/L. We multiplied the molar sum by the molecular weight of MECPP (308 g/mol) to express ∑DEHP as ng/ml.

**eTable 2.** Distribution of specific gravity normalized urinary phenol and phthalate biomarker concentrations from 384 mothers and 211 fathers in the Environment and Reproductive Health (EARTH) Study, 2005-2018.

| Windows/ | LOD (ng/ml) | Urine | % Detect ^b^ | SG-  Adjusted | SG-Adjusted | IQR |
| --- | --- | --- | --- | --- | --- | --- |
| Biomarker |  | N ^a^ |  | GM (GSD) ^c^ | Median | 25^th^ - 75^th^ |
|  |  |  |  |  | (ng/ml) | (ng/ml) |
| Maternal Preconception |  |  |  |  |  |  |
| MEHP | 0.5-1.2 | 1600 | 69.63 | 2.09 (0.09) | 1.92 | 1.19-3.54 |
| MEHHP | 0.2-0.7 | 1600 | 98.50 | 10.09 (0.50) | 9.16 | 5.34-19.30 |
| MEOHP | 0.2-0.7 | 1600 | 97.63 | 6.60 (0.32) | 5.89 | 3.49-11.78 |
| MECPP | 0.2-0.6 | 1600 | 91.50 | 17.77 (0.82) | 15.82 | 9.85-30.80 |
| MBP | 0.4-0.6 | 1600 | 95.75 | 9.65 (0.41) | 10.14 | 4.36-16.74 |
| MiBP | 0.2-0.3 | 1600 | 96.75 | 6.83 (0.30) | 6.76 | 4.12-11.88 |
| MBzP | 0.2-0.3 | 1600 | 89.38 | 2.93 (0.15) | 2.80 | 1.46-5.35 |
| MCPP | 0.1-0.2 | 1600 | 91.31 | 2.95 (0.15) | 2.86 | 1.464-5.74 |
| MCOP | 0.2-0.7 | 1600 | 98.44 | 20.11 (1.26) | 20.21 | 7.86-51.35 |
| MCNP | 0.2-0.6 | 1600 | 94.06 | 3.58 (0.16) | 3.46 | 1.95-5.98 |
| MEP | 0.4-0.8 | 1600 | 99.63 | 46.68 (2.84) | 41.81 | 20.55-90.15 |
| Bisphenol A | 0.1-0.4 | 1600 | 83.75 | 1.09 (0.04) | 1.05 | 0.72-1.59 |
| Methylparaben | 1.0 | 1600 | 99.50 | 113.9 (7.37) | 122.46 | 45.5-273.24 |
| Propylparaben | 0.1 | 1600 | 96.81 | 21.17 (1.80) | 27.15 | 7.34-71.20 |
| Butylparaben | 0.1 | 1600 | 62.81 | 0.93 (0.09) | 0.70 | 0.21-3.87 |
| Windows/ | LOD (ng/ml) | Urine | % Detect ^b^ | SG-Adjusted | SG-Adjusted | IQR |
| Biomarker |  | N ^a^ |  | GM (GSD) ^c^ | Median | 25^th^ - 75^th^ |
|  |  |  |  |  | (ng/ml) | (ng/ml) |
| Paternal Preconception |  |  |  |  |  |  |
| MEHP | 0.5-1.2 | 557 | 70.56 | 2.63 (0.21) | 2.39 | 1.18-5.63 |
| MEHHP | 0.2-0.7 | 557 | 98.74 | 13.92 (1.18) | 13.15 | 5.67-30.18 |
| MEOHP | 0.2-0.7 | 557 | 96.77 | 8.10 (0.69) | 7.90 | 3.73-17.35 |
| MECPP | 0.2-0.6 | 557 | 94.08 | 22.29 (1.84) | 22.61 | 9.60-46.86 |
| MBP | 0.4-0.6 | 557 | 95.69 | 9.27 (0.58) | 8.90 | 5.15-5.90 |
| MiBP | 0.2-0.3 | 557 | 96.23 | 6.85 (0.39) | 6.93 | 4.17-12.23 |
| MBzP | 0.2-0.3 | 557 | 93.72 | 2.90 (0.19) | 2.99 | 1.37-5.43 |
| MCPP | 0.1-0.2 | 557 | 94.08 | 3.55 (0.26) | 3.52 | 1.83-7.06 |
| MCOP | 0.2-0.7 | 557 | 98.20 | 22.41 (1.94) | 23.57 | 8.64-59.59 |
| MCNP | 0.2-0.6 | 557 | 95.15 | 3.88 (0.26) | 3.67 | 2.04-6.15 |
| MEP | 0.4-0.8 | 557 | 100.00 | 43.83 (3.86) | 42.06 | 17.33-98.29 |
| Bisphenol A | 0.1 | 557 | 87.79 | 1.34 (0.07) | 1.33 | 0.80-1.92 |
| Methylparaben | 1.0 | 557 | 98.74 | 26.77 (2.38) | 25.46 | 10.22-61.40 |
| Propylparaben | 0.1 | 557 | 89.95 | 2.88 (0.35) | 2.18 | 0.68-11.23 |
| Butylparaben | 0.1 | 557 | 32.14 | 0.26 (0.02) | 0.18 | 0.10-0.48 |

Note: monoethyl phthalate (MEP); mono-n-butyl phthalate (MBP); mono-isobutyl phthalate (MiBP); monobenzyl phthalate (MBzP); mono(2-ethylhexyl) phthalate (MEHP); mono(2-ethyl-5-hydroxyhexyl) phthalate (MEHHP); mono(2-ethyl-5-oxohexyl) phthalate (MEOHP); mono(2-ethyl-5-carboxypentyl) phthalate (MECPP); mono(3-carboxypropyl) phthalate (MCPP); monocarboxyisooctyl phthalate (MCOP); monocarboxyisononyl phthalate (MCNP).

^a^ Urine sample size.

^b^ Percentage of phenol concentrations above the limit of detection (ng/mL). All values below the LOD (<LOD) were assigned a value equal to the LOD divided by square root of two.

^c^ Geometric mean of urinary SG-adjusted concentrations expressed in ng/mL.

**eTable 3**. Loading scores of PCA-derived factors for **maternal** **preconception** phenol and phthalate mixtures among 384 mothers in the Environment and Reproductive Health (EARTH) Study, 2005 - 2018.

|  | DEHP and BPA Factor | Paraben Factor | High Molecular Weight Phthalate Factor | Low Molecular Weight Phthalate Factor |
| --- | --- | --- | --- | --- |
| Variance explained (%) | 33.21 | 16.08 | 13.38 | 10.17 |
| Eigenvalue | 4.982 | 2.41 | 2.01 | 1.52 |
| Loading score |  |  |  |  |
| MEOHP | 93* | 15 | 7 | 23 |
| MEHHP | 93* | 16 | 4 | 23 |
| MECPP | 92* | 16 | 17 | 11 |
| MEHP | 85* | 7 | -9 | 0 |
| BPA | 42 | 23 | 7 | 20 |
| MPB | 8 | 91* | 0 | 5 |
| PPB | 6 | 89* | 2 | 0 |
| BPB | 25 | 69* | 2 | -11 |
| MEP | 16 | 55* | 1 | 24 |
| MCOP | -14 | -2 | 90* | 14 |
| MCPP | 16 | 5 | 89* | 18 |
| MCNP | 13 | 3 | 85* | -6 |
| MiBP | 2 | 6 | 7 | 83* |
| MBP | 35 | 13 | 5 | 82* |
| MBzP | 19 | -4 | 11 | 72* |

Note: PCA, Principal component analysis; Loading scores were presented by multiplying 100 and rounding to the nearest integer. Values greater than 0.426717 are flagged by an '*'

Bisphenol A (BPA); methylparaben (MPB); propylparaben (PPB); butylparaben (BPB); monoethyl phthalate (MEP); mono-n-butyl phthalate (MBP); mono-isobutyl phthalate (MiBP); monobenzyl phthalate (MBzP); mono(2-ethylhexyl) phthalate (MEHP); mono(2-ethyl-5-hydroxyhexyl) phthalate (MEHHP); mono(2-ethyl-5-oxohexyl) phthalate (MEOHP); mono(2-ethyl-5-carboxypentyl) phthalate (MECPP); mono(3-carboxypropyl) phthalate (MCPP); monocarboxyisooctyl phthalate (MCOP); monocarboxyisononyl phthalate (MCNP).

**eTable 4**. Posterior inclusion probability in **maternal** **preconception** BKMR model among 384 mothers in the Environment and Reproductive Health (EARTH) Study, 2005 - 2018.

| Biomarker | Group (PCA factors) | GroupPIP^1^ | CondPIP^2^ |
| --- | --- | --- | --- |
| BPA | DEHP and BPA factor | 0.25 | 0.90 |
| MEHP | DEHP and BPA factor | 0.25 | 0.32 |
| MEOHP | DEHP and BPA factor | 0.25 | 0.03 |
| MEHHP | DEHP and BPA factor | 0.25 | 0.02 |
| MECPP | DEHP and BPA factor | 0.25 | 0.01 |
| MEP | Paraben factor | 0.11 | 0.93 |
| MPB | Paraben factor | 0.11 | 0.06 |
| BPB | Paraben factor | 0.11 | 0.01 |
| PPB | Paraben factor | 0.11 | 0.01 |
| MCPP | High molecular weight phthalate factor | 0.06 | 0.58 |
| MCNP | High molecular weight phthalate factor | 0.06 | 0.30 |
| MCOP | High molecular weight phthalate factor | 0.06 | 0.11 |
| MBP | Low molecular weight phthalate factor | 0.06 | 0.42 |
| MiBP | Low molecular weight phthalate factor | 0.06 | 0.35 |
| MBzP | Low molecular weight phthalate factor | 0.06 | 0.23 |

Note: Bisphenol A (BPA); methylparaben (MPB); propylparaben (PPB); butylparaben (BPB); monoethyl phthalate (MEP); mono-n-butyl phthalate (MBP); mono-isobutyl phthalate (MiBP); monobenzyl phthalate (MBzP); mono(2-ethylhexyl) phthalate (MEHP); mono(2-ethyl-5-hydroxyhexyl) phthalate (MEHHP); mono(2-ethyl-5-oxohexyl) phthalate (MEOHP); mono(2-ethyl-5-carboxypentyl) phthalate (MECPP); mono(3-carboxypropyl) phthalate (MCPP); monocarboxyisooctyl phthalate (MCOP); monocarboxyisononyl phthalate (MCNP); di-(2-ethylhexyl) phthalate (DEHP).

^1^ Group posterior inclusion probability: posterior probability of including the biomarker group

^2^ Conditional posterior inclusion probability: posterior probability of including the particular biomarker within the group.

**eTable 5**. Loading score of PCA-derived factors for **paternal** **preconception** phenol and phthalate biomarkers among 211 fathers in the Environment and Reproductive Health (EARTH) Study, 2005 - 2018.

|  | | DEHP and BPA Factor | High Molecular Weight Phthalate Factor | Low Molecular Weight Phthalate Factor | Paraben Factor |
| --- | --- | --- | --- | --- | --- |
| Variance explained (%) | 33.50 | | 15.40 | 13.93 | 10.03 |
| Eigenvalue | 5.02 | | 2.31 | 2.09 | 1.51 |
| Loading score |  | |  |  |  |
| MEHHP | 95* | | 3 | 22 | 3 |
| MEOHP | 94* | | 9 | 22 | 3 |
| MECPP | 93* | | 11 | 15 | 9 |
| MEHP | 86* | | -7 | 17 | 1 |
| BPA | 57* | | 28 | 5 | 4 |
| MCOP | -7 | | 89* | 18 | -10 |
| MCPP | 18 | | 89* | 23 | 4 |
| MCNP | 18 | | 81* | -7 | 19 |
| MiBP | 7 | | 14 | 82* | -3 |
| MBP | 41 | | 0 | 78* | 8 |
| MBzP | 19 | | 13 | 75* | 1 |
| MEP | 11 | | 5 | 35 | 28 |
| MPB | -4 | | 2 | 12 | 90* |
| PPB | -2 | | 4 | 15 | 89* |
| BPB | 13 | | 3 | -14 | 70* |

Note: PCA, Principal component analysis; Loading score values were multiplied by multiplying 100 and rounding to the nearest integer. Values greater than 0.426717 are flagged by an '*'

Bisphenol A (BPA); methylparaben (MPB); propylparaben (PPB); butylparaben (BPB); monoethyl phthalate (MEP); mono-n-butyl phthalate (MBP); mono-isobutyl phthalate (MiBP); monobenzyl phthalate (MBzP); mono(2-ethylhexyl) phthalate (MEHP); mono(2-ethyl-5-hydroxyhexyl) phthalate (MEHHP); mono(2-ethyl-5-oxohexyl) phthalate (MEOHP); mono(2-ethyl-5-carboxypentyl) phthalate (MECPP); mono(3-carboxypropyl) phthalate (MCPP); monocarboxyisooctyl phthalate (MCOP); monocarboxyisononyl phthalate (MCNP).

**eTable 6**. Posterior inclusion probability in **paternal** **preconception** BKMR model among 211 fathers in the Environment and Reproductive Health (EARTH) Study, 2005 - 2018.

| Biomarker | Group (PCA factors) | GroupPIP^1^ | CondPIP^2^ |
| --- | --- | --- | --- |
| MBP | Low molecular weight phthalate factor | 0.56 | 0.66 |
| MiBP | Low molecular weight phthalate factor | 0.56 | 0.30 |
| MBzP | Low molecular weight phthalate factor | 0.56 | 0.04 |
| MEOHP | DEHP and BPA factor | 0.27 | 0.41 |
| MEHHP | DEHP and BPA factor | 0.27 | 0.33 |
| MECPP | DEHP and BPA factor | 0.27 | 0.19 |
| MEHP | DEHP and BPA factor | 0.27 | 0.04 |
| BPA | DEHP and BPA factor | 0.27 | 0.03 |
| MCPP | High molecular weight phthalate factor | 0.07 | 0.50 |
| MCOP | High molecular weight phthalate factor | 0.07 | 0.33 |
| MCNP | High molecular weight phthalate factor | 0.07 | 0.17 |
| MPB | Paraben factor | 0.01 | 0.40 |
| PPB | Paraben factor | 0.01 | 0.34 |
| MEP | Paraben factor | 0.01 | 0.14 |
| BPB | Paraben factor | 0.01 | 0.11 |

Note: Bisphenol A (BPA); methylparaben (MPB); propylparaben (PPB); butylparaben (BPB); monoethyl phthalate (MEP); mono-n-butyl phthalate (MBP); mono-isobutyl phthalate (MiBP); monobenzyl phthalate (MBzP); mono(2-ethylhexyl) phthalate (MEHP); mono(2-ethyl-5-hydroxyhexyl) phthalate (MEHHP); mono(2-ethyl-5-oxohexyl) phthalate (MEOHP); mono(2-ethyl-5-carboxypentyl) phthalate (MECPP); mono(3-carboxypropyl) phthalate (MCPP); monocarboxyisooctyl phthalate (MCOP); monocarboxyisononyl phthalate (MCNP); di-(2-ethylhexyl) phthalate (DEHP).

^1^ Group posterior inclusion probability: posterior probability of including the biomarker group

^2^ Conditional posterior inclusion probability: posterior probability of including the particular biomarker within the group.

**eTable 7**. Posterior inclusion probability in **couple's preconception** BKMR model among 203 couples in the Environment and Reproductive Health (EARTH) Study, 2005 - 2018.

| Biomarker | Group | GroupPIP^1^ | CondPIP^2^ |
| --- | --- | --- | --- |
| MBP | Paternal | 0.72 | 0.41 |
| MEOHP | Paternal | 0.72 | 0.15 |
| MEHHP | Paternal | 0.72 | 0.13 |
| MECPP | Paternal | 0.72 | 0.09 |
| MiBP | Paternal | 0.72 | 0.08 |
| MBzP | Paternal | 0.72 | 0.06 |
| MEHP | Paternal | 0.72 | 0.03 |
| BPA | Paternal | 0.72 | 0.01 |
| MCNP | Paternal | 0.72 | 0.01 |
| MCPP | Paternal | 0.72 | 0.01 |
| MEP | Paternal | 0.72 | 0.00 |
| BPB | Paternal | 0.72 | 0.00 |
| MCOP | Paternal | 0.72 | 0.00 |
| MPB | Paternal | 0.72 | 0.00 |
| PPB | Paternal | 0.72 | 0.00 |
| MBP | Maternal | 0.18 | 0.20 |
| MEP | Maternal | 0.18 | 0.17 |
| BPA | Maternal | 0.18 | 0.13 |
| MCPP | Maternal | 0.18 | 0.12 |
| MEHP | Maternal | 0.18 | 0.09 |
| MCNP | Maternal | 0.18 | 0.08 |
| MPB | Maternal | 0.18 | 0.05 |
| MBzP | Maternal | 0.18 | 0.04 |
| MiBP | Maternal | 0.18 | 0.03 |
| MECPP | Maternal | 0.18 | 0.03 |
| MCOP | Maternal | 0.18 | 0.02 |
| MEOHP | Maternal | 0.18 | 0.02 |
| BPB | Maternal | 0.18 | 0.01 |
| MEHHP | Maternal | 0.18 | 0.01 |
| PPB | Maternal | 0.18 | 0.01 |

Note: Bisphenol A (BPA); methylparaben (MPB); propylparaben (PPB); butylparaben (BPB); monoethyl phthalate (MEP); mono-n-butyl phthalate (MBP); mono-isobutyl phthalate (MiBP); monobenzyl phthalate (MBzP); mono(2-ethylhexyl) phthalate (MEHP); mono(2-ethyl-5-hydroxyhexyl) phthalate (MEHHP); mono(2-ethyl-5-oxohexyl) phthalate (MEOHP); mono(2-ethyl-5-carboxypentyl) phthalate (MECPP); mono(3-carboxypropyl) phthalate (MCPP); monocarboxyisooctyl phthalate (MCOP); monocarboxyisononyl phthalate (MCNP).

^1^ Group posterior inclusion probability: posterior probability of including the biomarker group

^2^ Conditional posterior inclusion probability: posterior probability of including the particular biomarker within the group.

**eTable 8**. Adjusted difference (95% CI) in birthweight (gram) by PCA-derived factors scores from 384 mothers and 211 fathers, after further **adjustment for gestational age**.

| PCA-derived factors | Maternal | | Paternal | |  |
| --- | --- | --- | --- | --- | --- |
|  | Beta (95% CI) ^a^ | P values | Beta (95% CI) ^b^ | P values |  |
| DEHP and BPA factor | -0.64 (-42.81, 41.53) | 0.98 | -49.33 (-111.55, 12.90) | 0.12 |  |
| Paraben factor | -10.63 (-53.18, 31.92) | 0.62 | 47.74 (-12.51, 108.00) | 0.12 |  |
| High molecular weight phthalate factor | -13.18 (-56.12, 29.76) | 0.55 | -52.60 (-111.99, 6.79) | 0.08 |  |
| Low molecular weight phthalate factor | -41.63 (-83.91, 0.65) | 0.05 | -82.31 (-142.66, -21.95) | 0.008 |  |

Note: PCA, Principal component analysis; di-(2-ethylhexyl) phthalate (DEHP).

^a^ adjusted for maternal age (continuous), BMI (continuous), ART (yes/no), smoking (ever/never), education (categorical), races (categorical), and gestational age (continuous).

^b^ adjusted for maternal and paternal age (continuous), maternal and paternal BMI (continuous), maternal and paternal smoking (ever/never), maternal education (categorical), maternal races (categorical), ART (yes/no), and gestational age (continuous).

**eTable 9**. Adjusted difference (95% CI) in birthweight (gram) by PCA-derived factors scores from **203 mothers** who enrolled with a male partner.

| PCA-derived factors | Beta (95% CI) ^a^ | P values |  |
| --- | --- | --- | --- |
| DEHP and BPA factor | -2.57 (-70.02, 64.88) | 0.94 |  |
| Paraben factor | -21.71 (-89.38, 45.96) | 0.53 |  |
| High molecular weight phthalate factor | 6.53 (-65.09, 78.14) | 0.86 |  |
| Low molecular weight phthalate factor | -52.36 (-121.83, 17.11) | 0.14 |  |

Note: PCA, Principal component analysis; di-(2-ethylhexyl) phthalate (DEHP).

^a^ adjusted for maternal age (continuous), BMI (continuous), ART (yes/no), smoking (ever/never), education (categorical), races (categorical).

**eTable 10**. Posterior inclusion probability in maternal preconception BKMR model restricted to **203 mothers** who enrolled with a male partner.

| Biomarker | Group (PCA factors) | GroupPIP^1^ | CondPIP^2^ |
| --- | --- | --- | --- |
| MBP | Low molecular weight phthalate factor | 0.21 | 0.78 |
| MBzP | Low molecular weight phthalate factor | 0.21 | 0.14 |
| MiBP | Low molecular weight phthalate factor | 0.21 | 0.09 |
| MCPP | High molecular weight phthalate factor | 0.16 | 0.67 |
| MCNP | High molecular weight phthalate factor | 0.16 | 0.24 |
| MCOP | High molecular weight phthalate factor | 0.16 | 0.09 |
| MEP | Paraben factor | 0.14 | 0.81 |
| MPB | Paraben factor | 0.14 | 0.14 |
| BPB | Paraben factor | 0.14 | 0.03 |
| PPB | Paraben factor | 0.14 | 0.02 |
| BPA | DEHP and BPA factor | 0.09 | 0.70 |
| MEHP | DEHP and BPA factor | 0.09 | 0.14 |
| MECPP | DEHP and BPA factor | 0.09 | 0.09 |
| MEHHP | DEHP and BPA factor | 0.09 | 0.05 |
| MEOHP | DEHP and BPA factor | 0.09 | 0.02 |

Note: Bisphenol A (BPA); methylparaben (MPB); propylparaben (PPB); butylparaben (BPB); monoethyl phthalate (MEP); mono-n-butyl phthalate (MBP); mono-isobutyl phthalate (MiBP); monobenzyl phthalate (MBzP); mono(2-ethylhexyl) phthalate (MEHP); mono(2-ethyl-5-hydroxyhexyl) phthalate (MEHHP); mono(2-ethyl-5-oxohexyl) phthalate (MEOHP); mono(2-ethyl-5-carboxypentyl) phthalate (MECPP); mono(3-carboxypropyl) phthalate (MCPP); monocarboxyisooctyl phthalate (MCOP); monocarboxyisononyl phthalate (MCNP); di-(2-ethylhexyl) phthalate (DEHP).

^1^ Group posterior inclusion probability: posterior probability of including the biomarker group

^2^ Conditional posterior inclusion probability: posterior probability of including the particular biomarker within the group.

**eFigure 1**. Spearman correlation plot for **maternal** **preconception** phenol and phthalate biomarkers among 384 mothers in the Environment and Reproductive Health (EARTH) Study, 2005 - 2018.

Note: Bisphenol A (BPA); methylparaben (MPB); propylparaben (PPB); butylparaben (BPB); monoethyl phthalate (MEP); mono-n-butyl phthalate (MBP); mono-isobutyl phthalate (MiBP); monobenzyl phthalate (MBzP); mono(2-ethylhexyl) phthalate (MEHP); mono(2-ethyl-5-hydroxyhexyl) phthalate (MEHHP); mono(2-ethyl-5-oxohexyl) phthalate (MEOHP); mono(2-ethyl-5-carboxypentyl) phthalate (MECPP); mono(3-carboxypropyl) phthalate (MCPP); monocarboxyisooctyl phthalate (MCOP); monocarboxyisononyl phthalate (MCNP).

**eFigure 2**. Spearman correlation plot for **paternal** **preconception** phenol and phthalate biomarkers among 211 fathers in the Environment and Reproductive Health (EARTH) Study, 2005 - 2018.

Note: Bisphenol A (BPA); methylparaben (MPB); propylparaben (PPB); butylparaben (BPB); monoethyl phthalate (MEP); mono-n-butyl phthalate (MBP); mono-isobutyl phthalate (MiBP); monobenzyl phthalate (MBzP); mono(2-ethylhexyl) phthalate (MEHP); mono(2-ethyl-5-hydroxyhexyl) phthalate (MEHHP); mono(2-ethyl-5-oxohexyl) phthalate (MEOHP); mono(2-ethyl-5-carboxypentyl) phthalate (MECPP); mono(3-carboxypropyl) phthalate (MCPP); monocarboxyisooctyl phthalate (MCOP); monocarboxyisononyl phthalate (MCNP).

**eFigure 3**. Spearman correlation plot for **couples’** **preconception** phenol and phthalate biomarkers among 203 couples in the Environment and Reproductive Health (EARTH) Study, 2005 - 2018.

Note: “Fem_” denotes maternal biomarkers; “ma_” denotes paternal biomarkers.

Bisphenol A (BPA); methylparaben (MPB); propylparaben (PPB); butylparaben (BPB); monoethyl phthalate (MEP); mono-n-butyl phthalate (MBP); mono-isobutyl phthalate (MiBP); monobenzyl phthalate (MBzP); mono(2-ethylhexyl) phthalate (MEHP); mono(2-ethyl-5-hydroxyhexyl) phthalate (MEHHP); mono(2-ethyl-5-oxohexyl) phthalate (MEOHP); mono(2-ethyl-5-carboxypentyl) phthalate (MECPP); mono(3-carboxypropyl) phthalate (MCPP); monocarboxyisooctyl phthalate (MCOP); monocarboxyisononyl phthalate (MCNP).

**eFigure 4.** Change (estimates and credible intervals) in birthweight (gram) comparing the **maternal** **preconception** biomarker of interest at the 75^th^ *vs.* 25^th^ percentile, when setting the remaining maternal biomarkers at their 25^th^, 50^th^ and 75^th^ percentiles among 384 mothers.

**
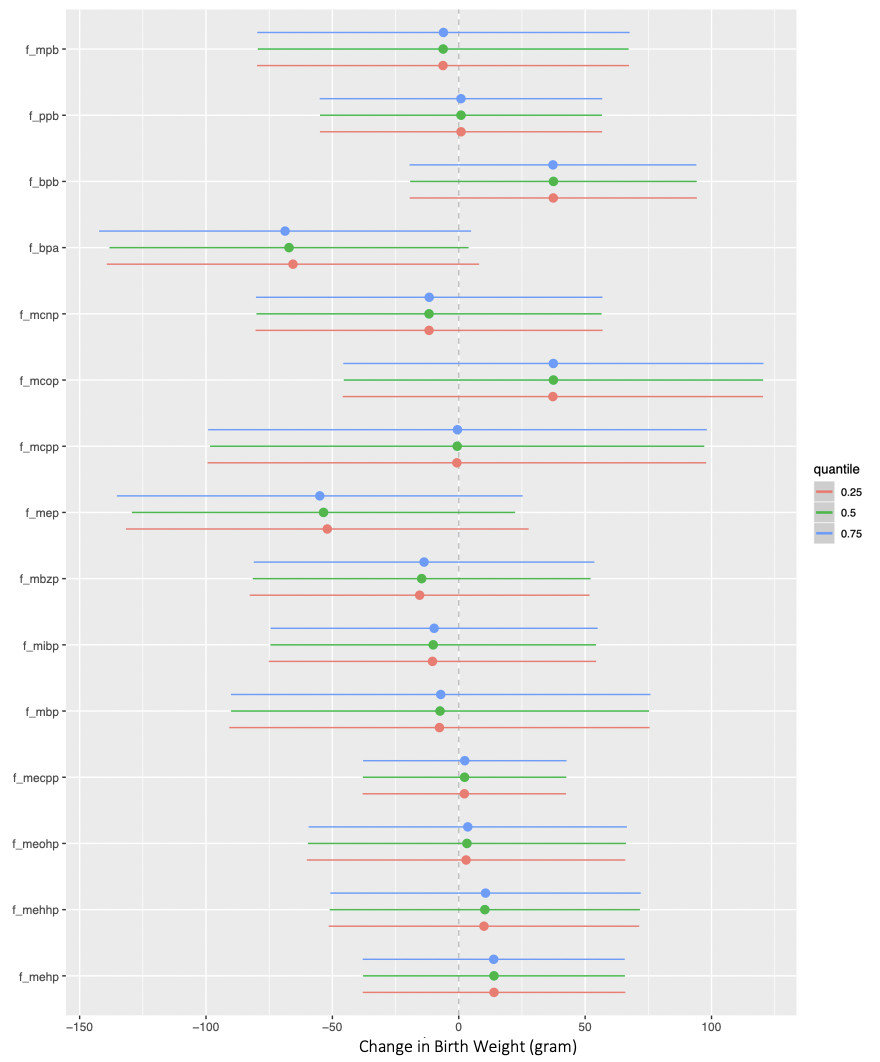
**

Note. “f_” denotes female or maternal preconception biomarker concentration; bisphenol A (BPA); methylparaben (MPB); propylparaben (PPB); butylparaben (BPB); monoethyl phthalate (MEP); mono-n-butyl phthalate (MBP); mono-isobutyl phthalate (MiBP); monobenzyl phthalate (MBzP); mono(2-ethylhexyl) phthalate (MEHP); mono(2-ethyl-5-hydroxyhexyl) phthalate (MEHHP); mono(2-ethyl-5-oxohexyl) phthalate (MEOHP); mono(2-ethyl-5-carboxypentyl) phthalate (MECPP); mono(3-carboxypropyl) phthalate (MCPP); monocarboxyisooctyl phthalate (MCOP); monocarboxyisononyl phthalate (MCNP); models were adjusted for maternal age (continuous), BMI (continuous), ART (yes/no), smoking (ever/never), education (categorical), races (categorical).

**eFigure 5.** Joint effect on birthweight comparing each 5^th^ quantile change in **maternal preconception** mixture concentrations from the 25^th^ to 75^th^ quantile to the median concentration among 384 mothers in the Environment and Reproductive Health (EARTH) Study, 2005 - 2018.


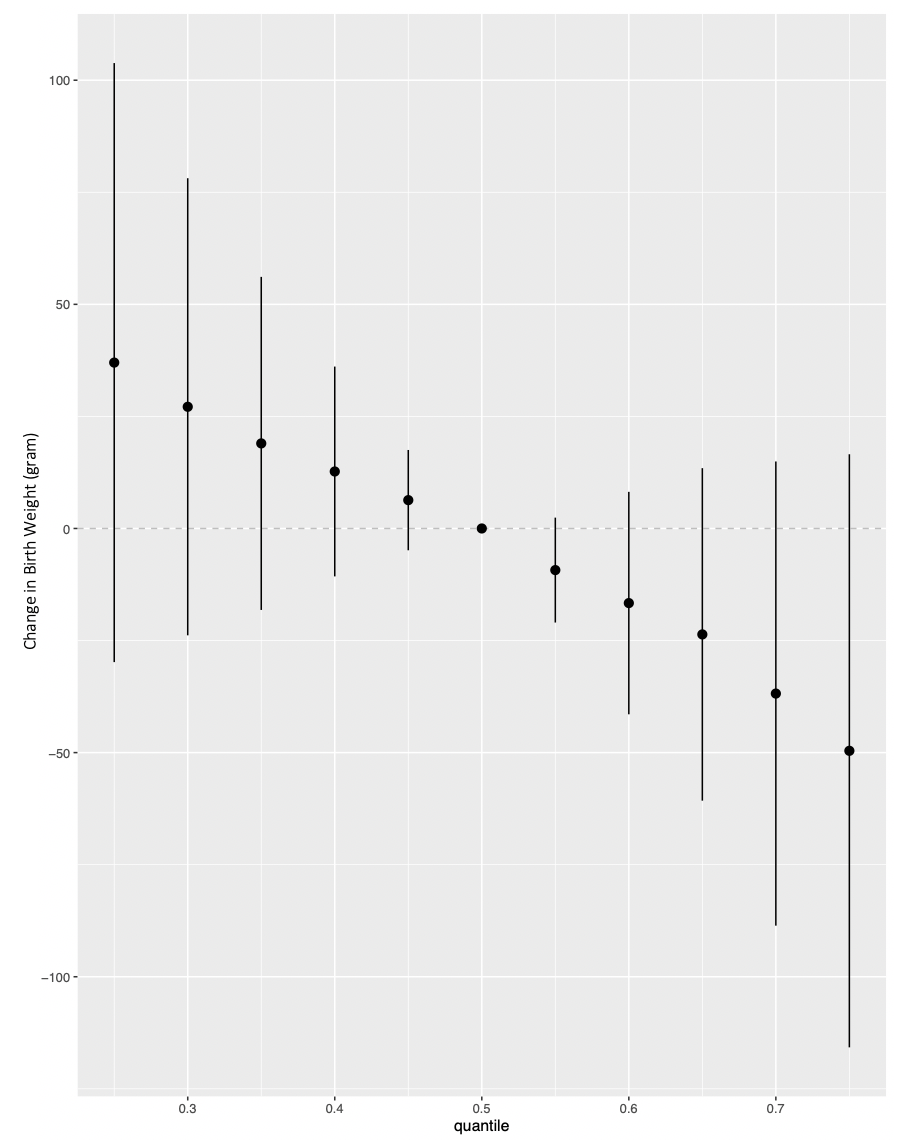


Note: models were adjusted for maternal age (continuous), BMI (continuous), ART (yes/no), smoking (ever/never), education (categorical), races (categorical).

**eFigure 6.** Change (estimates and credible intervals) in birthweight (gram) comparing the **paternal** **preconception** biomarker of interest at the 75^th^ *vs.* 25^th^ percentile, when setting the remaining paternal biomarkers at their 25^th^, 50^th^ and 75^th^ percentiles among 211 fathers.

**
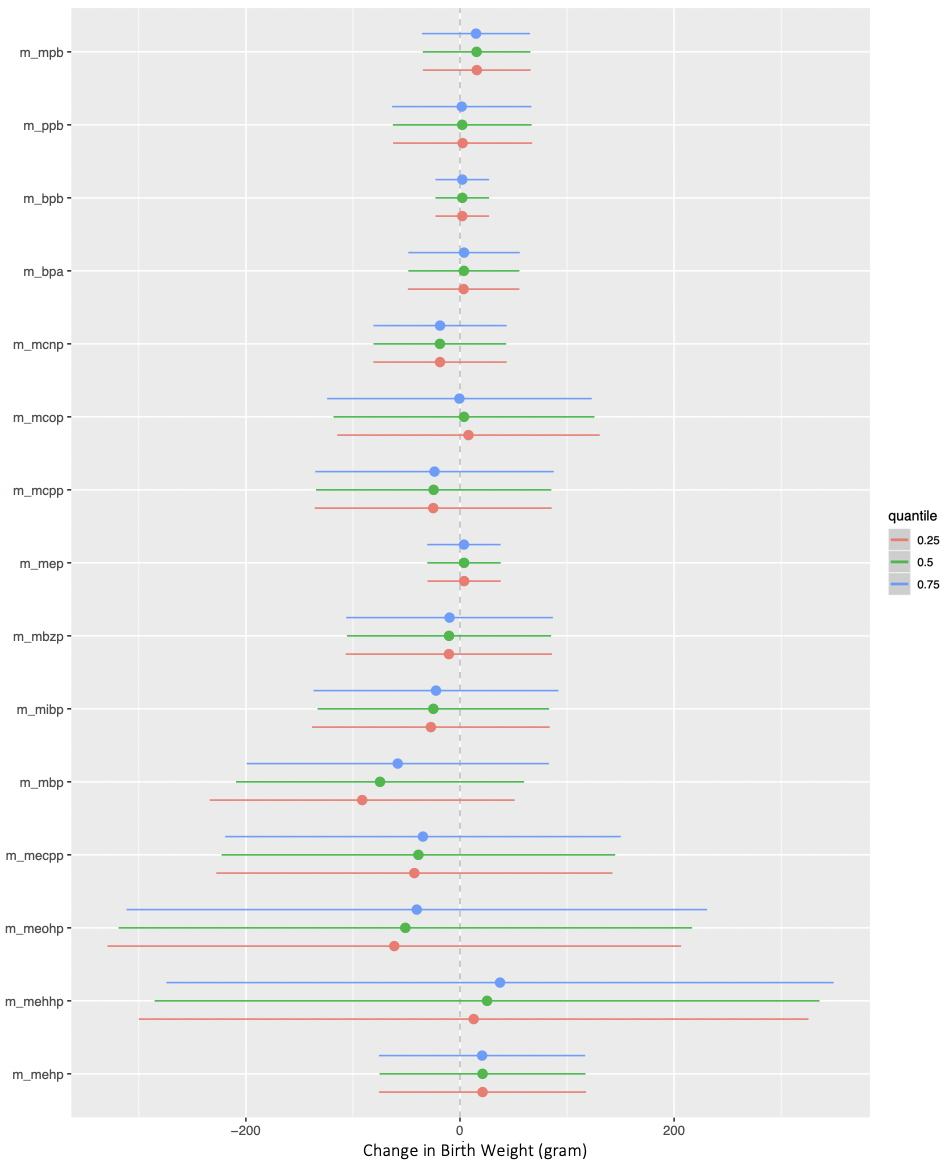
**

Note: “m_” denotes male or paternal preconception biomarker concentration; bisphenol A (BPA); methylparaben (MPB); propylparaben (PPB); butylparaben (BPB); monoethyl phthalate (MEP); mono-n-butyl phthalate (MBP); mono-isobutyl phthalate (MiBP); monobenzyl phthalate (MBzP); mono(2-ethylhexyl) phthalate (MEHP); mono(2-ethyl-5-hydroxyhexyl) phthalate (MEHHP); mono(2-ethyl-5-oxohexyl) phthalate (MEOHP); mono(2-ethyl-5-carboxypentyl) phthalate (MECPP); mono(3-carboxypropyl) phthalate (MCPP); monocarboxyisooctyl phthalate (MCOP); monocarboxyisononyl phthalate (MCNP); models were adjusted for maternal and paternal age (continuous), maternal and paternal BMI (continuous), maternal and paternal smoking (ever/never), maternal education (categorical), maternal races (categorical), ART (yes/no).

**eFigure 7.** Change (estimates and credible intervals) in birthweight (gram) comparing **couples’** **preconception** biomarker of interest at the 75^th^ *vs.* 25^th^ percentile, when setting the remaining couples’ biomarkers at their 25^th^, 50^th^ and 75^th^ percentiles among 203 couples.

**
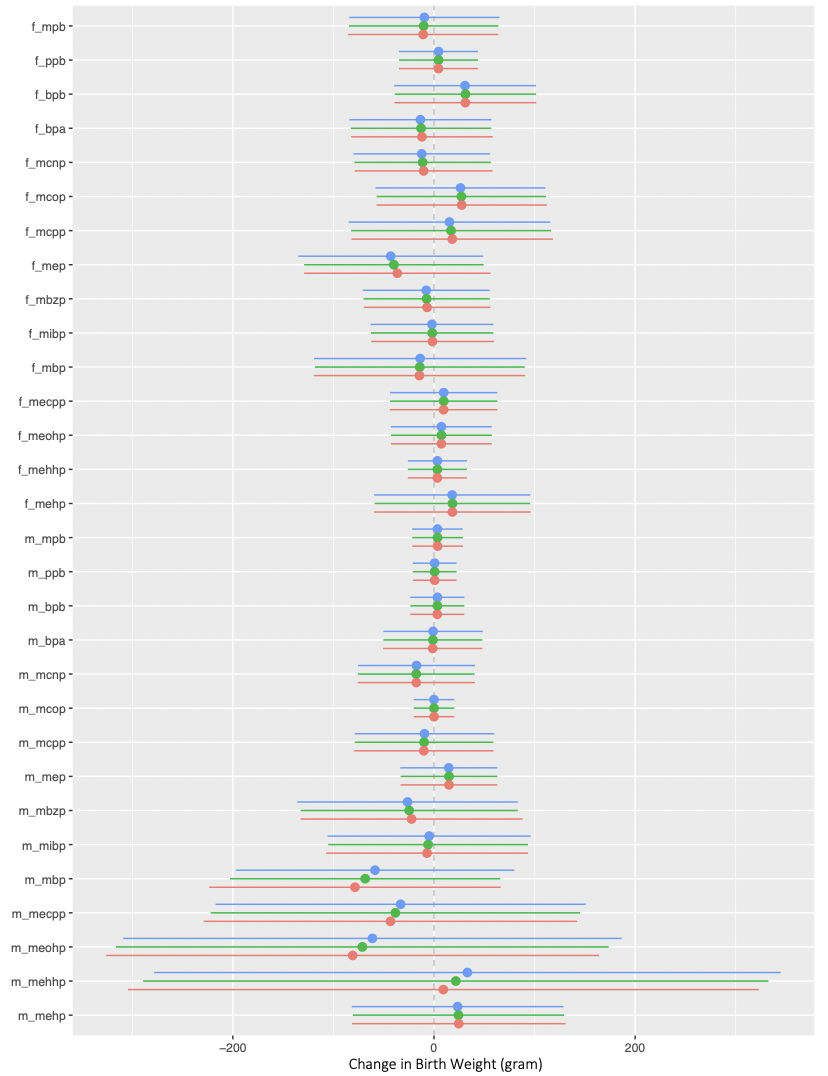
**

Note: “f_” denotes female or maternal preconception biomarker concentration; “m_” denotes male or paternal preconception biomarker concentration; bisphenol A (BPA); methylparaben (MPB); propylparaben (PPB); butylparaben (BPB); monoethyl phthalate (MEP); mono-n-butyl phthalate (MBP); mono-isobutyl phthalate (MiBP); monobenzyl phthalate (MBzP); mono(2-ethylhexyl) phthalate (MEHP); mono(2-ethyl-5-hydroxyhexyl) phthalate (MEHHP); mono(2-ethyl-5-oxohexyl) phthalate (MEOHP); mono(2-ethyl-5-carboxypentyl) phthalate (MECPP); mono(3-carboxypropyl) phthalate (MCPP); monocarboxyisooctyl phthalate (MCOP); monocarboxyisononyl phthalate (MCNP); models were adjusted for maternal and paternal age (continuous), maternal and paternal BMI (continuous), maternal and paternal smoking (ever/never), maternal education (categorical), maternal races (categorical), ART (yes/no).

**eFigure 8.** Dose-response associations of individual **maternal preconception** phenol and phthalate metabolite concentrations on birthweight, holding other biomarkers at their median concentrations after further **adjusting for gestational age** among 384 mothers.


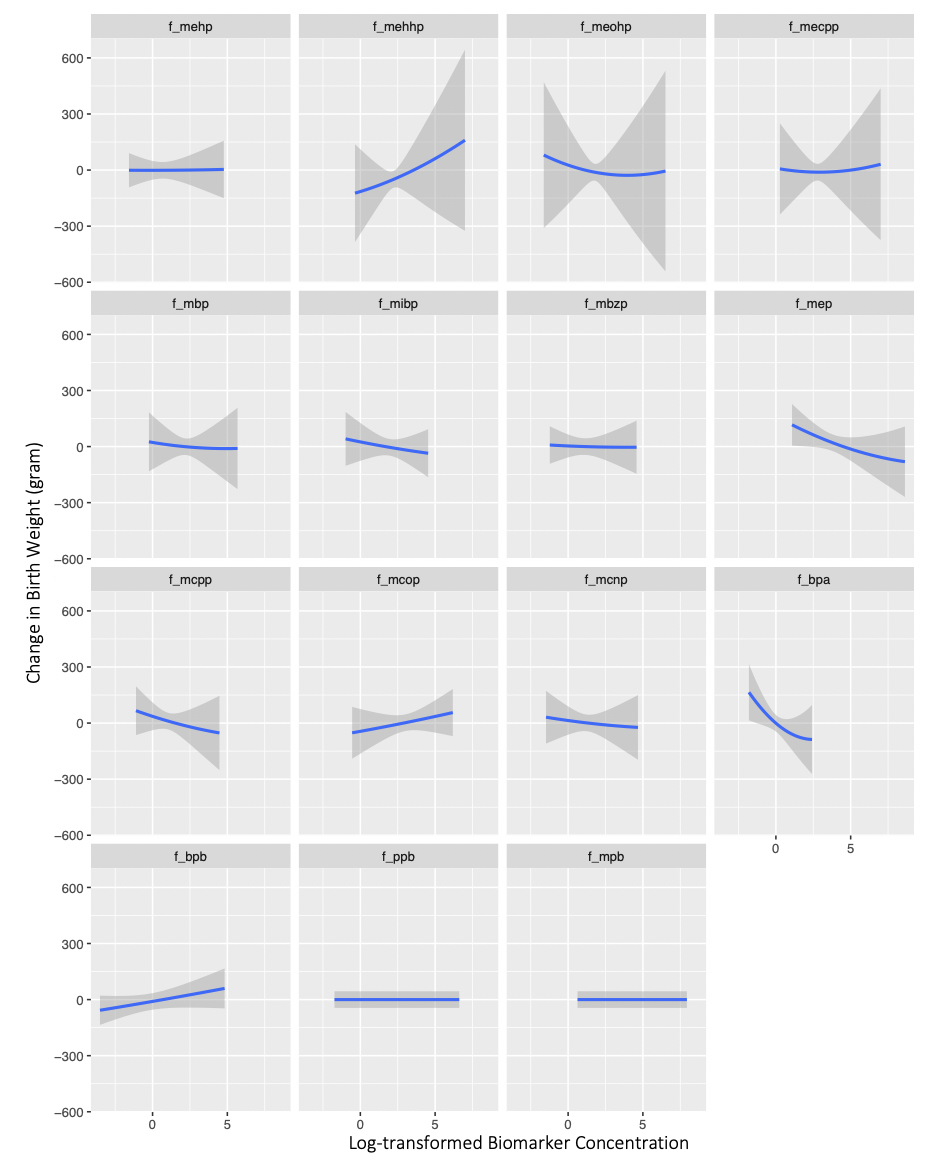


Note: “f_” denotes female or maternal preconception biomarker concentration; bisphenol A (BPA); methylparaben (MPB); propylparaben (PPB); butylparaben (BPB); monoethyl phthalate (MEP); mono-n-butyl phthalate (MBP); mono-isobutyl phthalate (MiBP); monobenzyl phthalate (MBzP); mono(2-ethylhexyl) phthalate (MEHP); mono(2-ethyl-5-hydroxyhexyl) phthalate (MEHHP); mono(2-ethyl-5-oxohexyl) phthalate (MEOHP); mono(2-ethyl-5-carboxypentyl) phthalate (MECPP); mono(3-carboxypropyl) phthalate (MCPP); monocarboxyisooctyl phthalate (MCOP); monocarboxyisononyl phthalate (MCNP); models were adjusted for maternal age (continuous), BMI (continuous), ART (yes/no), smoking (ever/never), education (categorical), races (categorical), and gestational age (continuous).

**eFigure 9.** Joint effect on comparing each 5^th^ quantile change in **maternal preconception** mixture concentrations from the 25^th^ to 75^th^ quantile to the median concentration, **adjusting for gestational age** among 384 mothers.


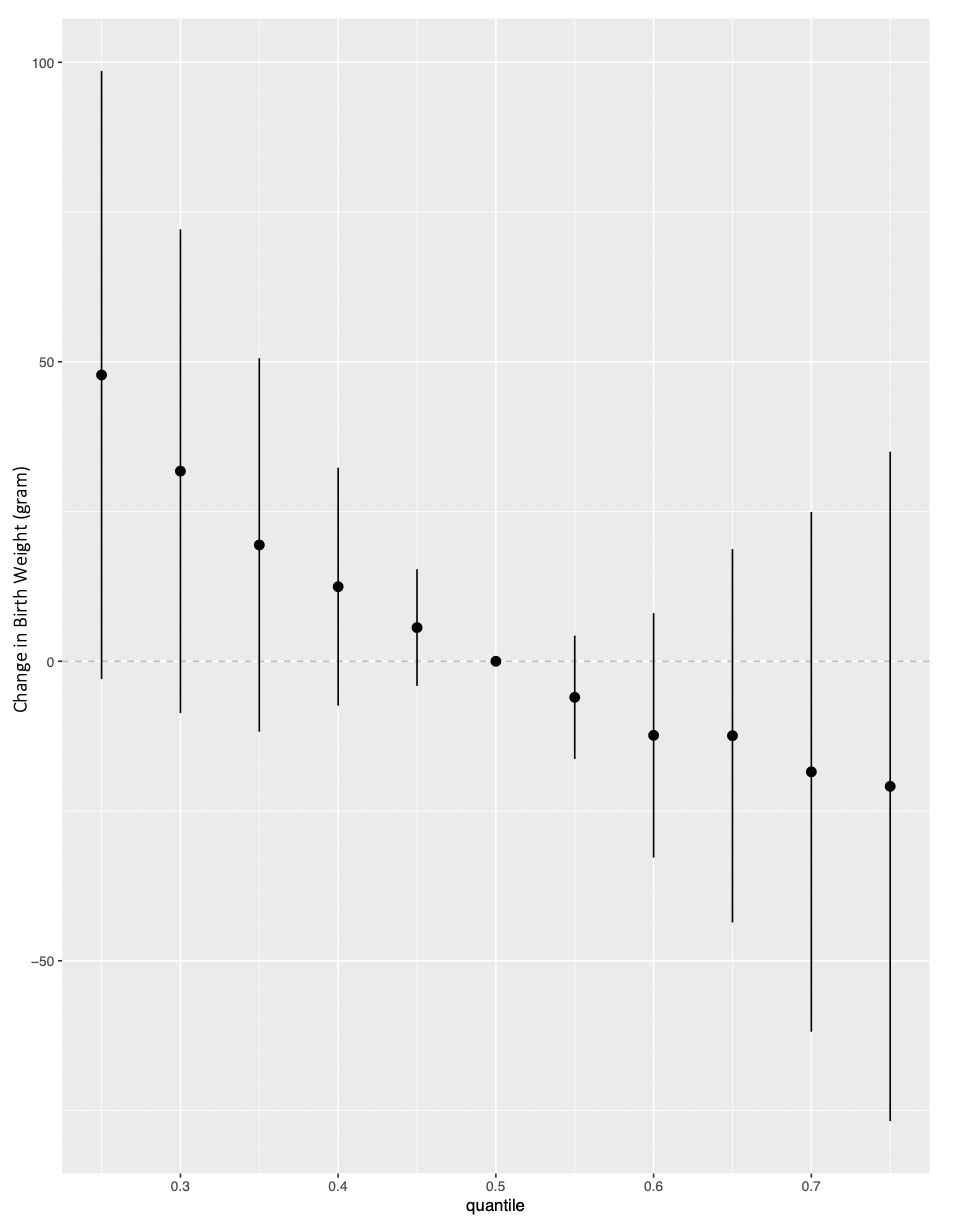


Note: models were adjusted for maternal age (continuous), BMI (continuous), ART (yes/no), smoking (ever/never), education (categorical), races (categorical), and gestational age (continuous).

**eFigure 10.** Dose-response associations of individual **paternal** **preconception** phenol and phthalate metabolite concentrations on birthweight, holding all other biomarkers at their median concentrations, after further **adjusting for gestational age** among 211 fathers.


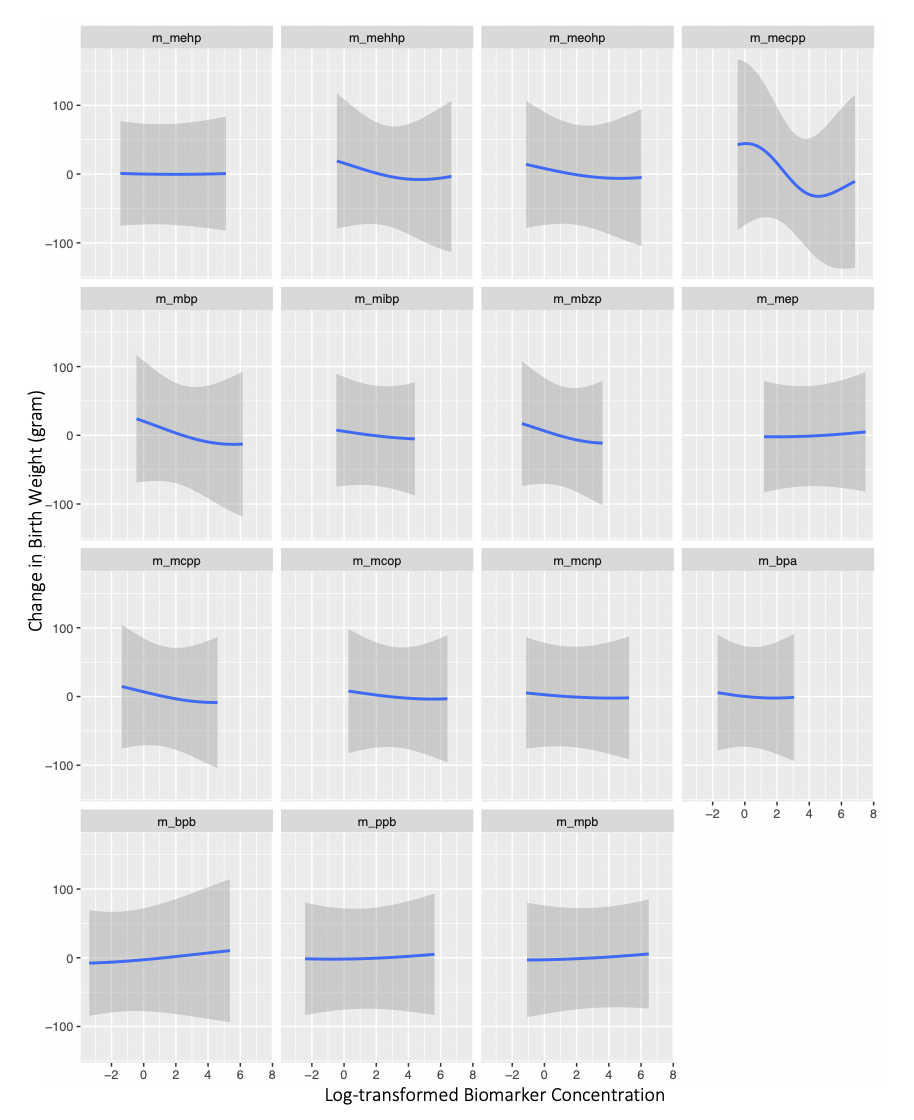


Note: “m_” denotes male or paternal preconception biomarker concentration; bisphenol A (BPA); methylparaben (MPB); propylparaben (PPB); butylparaben (BPB); monoethyl phthalate (MEP); mono-n-butyl phthalate (MBP); mono-isobutyl phthalate (MiBP); monobenzyl phthalate (MBzP); mono(2-ethylhexyl) phthalate (MEHP); mono(2-ethyl-5-hydroxyhexyl) phthalate (MEHHP); mono(2-ethyl-5-oxohexyl) phthalate (MEOHP); mono(2-ethyl-5-carboxypentyl) phthalate (MECPP); mono(3-carboxypropyl) phthalate (MCPP); monocarboxyisooctyl phthalate (MCOP); monocarboxyisononyl phthalate (MCNP); models were adjusted for maternal and paternal age (continuous), maternal and paternal BMI (continuous), maternal and paternal smoking (ever/never), maternal education (categorical), maternal races (categorical), ART (yes/no), and gestational age (continuous).

**eFigure 11.** Joint effect on comparing each 5^th^ quantile change in **paternal preconception** mixture concentrations from the 25^th^ to 75^th^ quantile to the median concentration, **adjusting for gestational age** among 211 fathers.


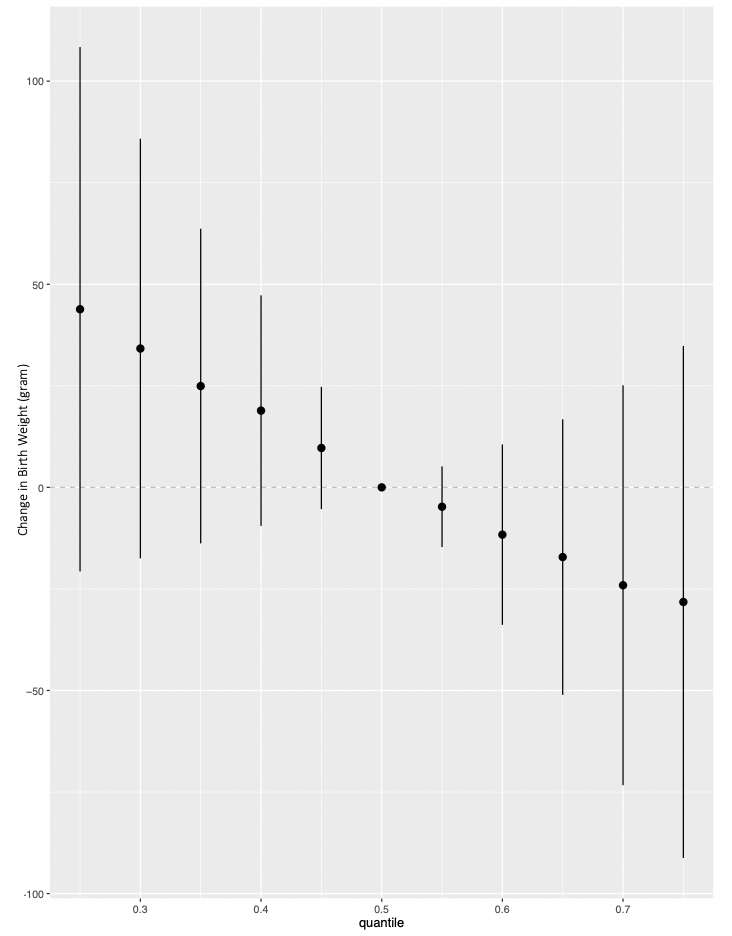


Note: Models were adjusted for maternal and paternal age (continuous), maternal and paternal BMI (continuous), maternal and paternal smoking (ever/never), maternal education (categorical), maternal races (categorical), ART (yes/no), and gestational age (continuous).

**eFigure 12.** Dose-response associations of **couple’s individual preconception** phenol and phthalate metabolite concentrations on birthweight, holding all other biomarkers at their median concentrations, after further **adjusting for gestational age** among 203 couples.


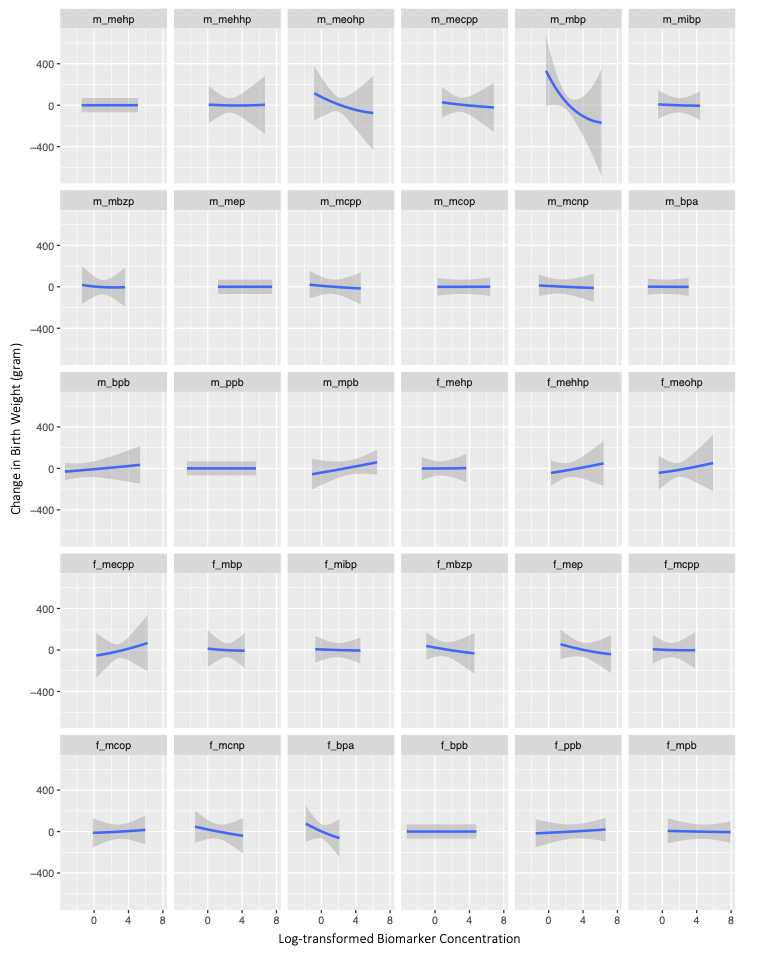


Note: “f_” denotes female or maternal preconception biomarker concentration; “m_” denotes male or paternal preconception biomarker concentration; bisphenol A (BPA); methylparaben (MPB); propylparaben (PPB); butylparaben (BPB); monoethyl phthalate (MEP); mono-n-butyl phthalate (MBP); mono-isobutyl phthalate (MiBP); monobenzyl phthalate (MBzP); mono(2-ethylhexyl) phthalate (MEHP); mono(2-ethyl-5-hydroxyhexyl) phthalate (MEHHP); mono(2-ethyl-5-oxohexyl) phthalate (MEOHP); mono(2-ethyl-5-carboxypentyl) phthalate (MECPP); mono(3-carboxypropyl) phthalate (MCPP); monocarboxyisooctyl phthalate (MCOP); monocarboxyisononyl phthalate (MCNP); models were adjusted for maternal and paternal age (continuous), maternal and paternal BMI (continuous), maternal and paternal smoking (ever/never), maternal education (categorical), maternal races (categorical), ART (yes/no), and gestational age (continuous).

**eFigure 13.** Joint effect on birthweight comparing each 5^th^ quantile change in **couples’ preconception** mixture concentrations from the 25^th^ to 75^th^ quantile to the median concentration, **adjusting for gestational age** among 203 couples.


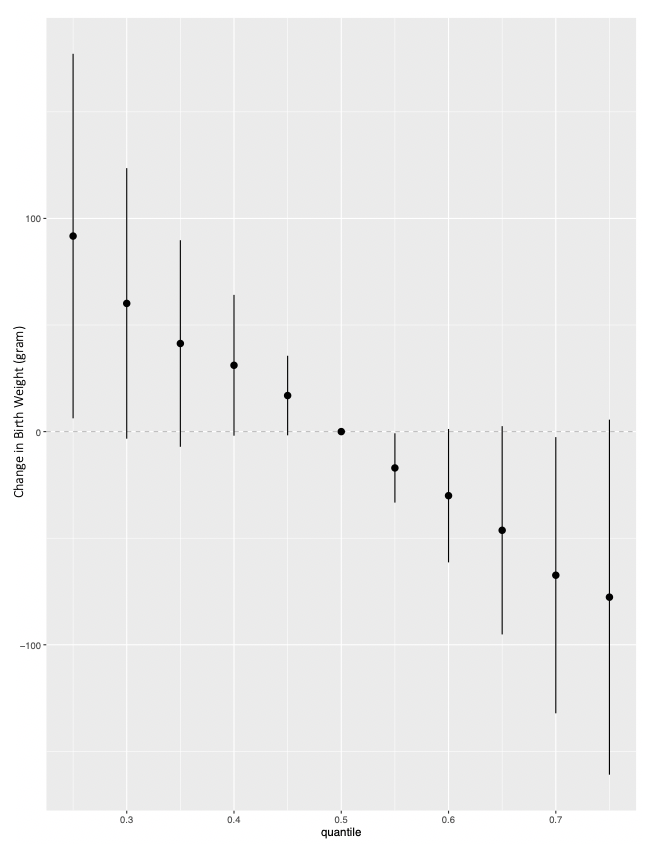


Note: Models were adjusted for maternal and paternal age (continuous), maternal and paternal BMI (continuous), maternal and paternal smoking (ever/never), maternal education (categorical), maternal races (categorical), ART (yes/no), and gestational age (continuous).

**eFigure 14**. Dose-response associations of individual **maternal** **preconception** phenol and phthalate metabolite concentrations on birthweight, holding all other biomarkers at their median concentrations among **203 mothers** who enrolled as couples.


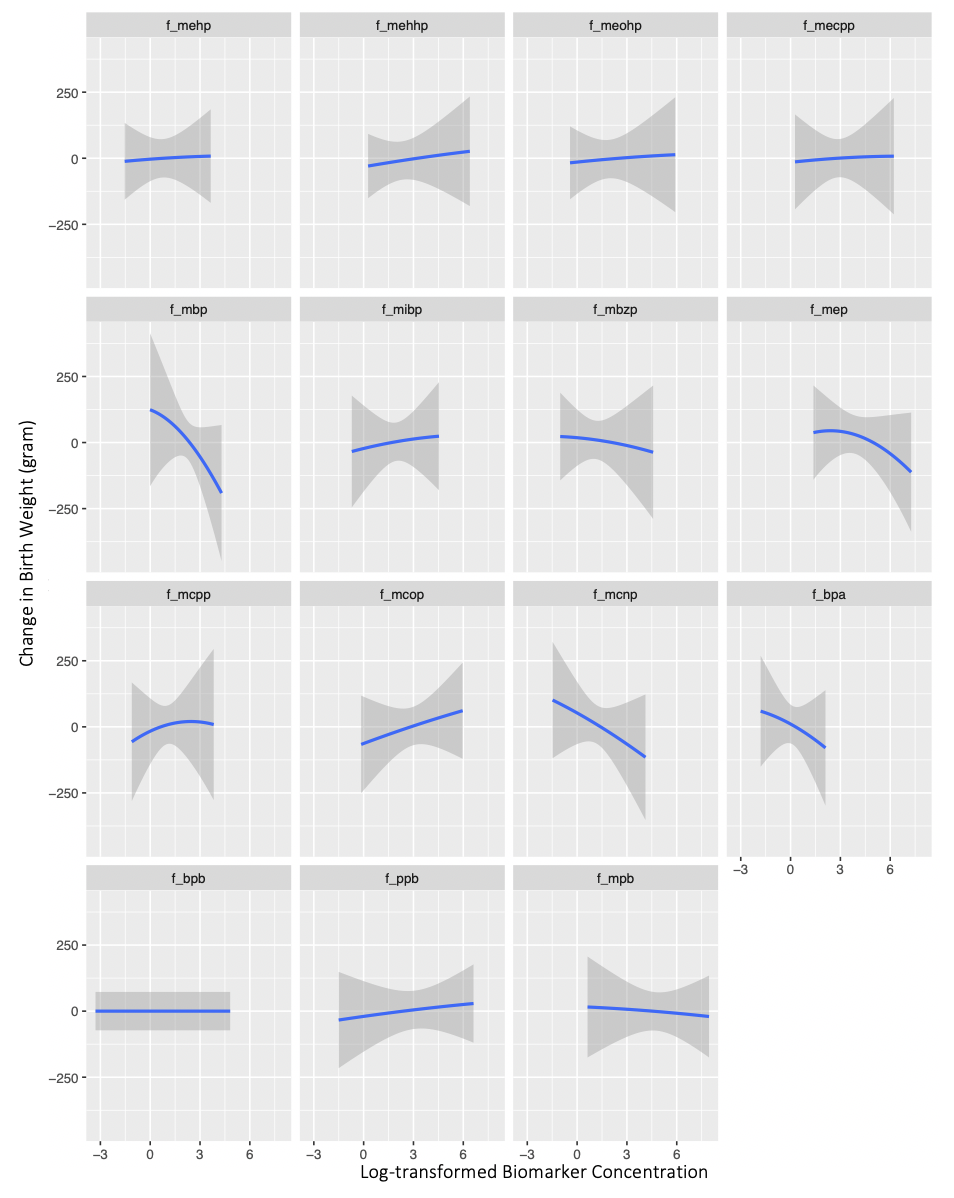


Note: “f_” denotes female or maternal preconception biomarker concentration; bisphenol A (BPA); methylparaben (MPB); propylparaben (PPB); butylparaben (BPB); monoethyl phthalate (MEP); mono-n-butyl phthalate (MBP); mono-isobutyl phthalate (MiBP); monobenzyl phthalate (MBzP); mono(2-ethylhexyl) phthalate (MEHP); mono(2-ethyl-5-hydroxyhexyl) phthalate (MEHHP); mono(2-ethyl-5-oxohexyl) phthalate (MEOHP); mono(2-ethyl-5-carboxypentyl) phthalate (MECPP); mono(3-carboxypropyl) phthalate (MCPP); monocarboxyisooctyl phthalate (MCOP); monocarboxyisononyl phthalate (MCNP); models were adjusted for maternal age (continuous), BMI (continuous), ART (yes/no), smoking (ever/never), education (categorical), races (categorical).
